# A practice-oriented guide to statistical inference in linear modeling for non-normal or heteroskedastic error distributions

**DOI:** 10.3758/s13428-025-02801-4

**Published:** 2025-11-10

**Authors:** Hanna Rajh-Weber, Stefan Ernest Huber, Martin Arendasy

**Affiliations:** Department of Psychology, University of Graz, Universitätsplatz 2, 8010 Graz, Austria

**Keywords:** Linear regression, Violated assumptions, Bootstrap, Robust inference, Simulation

## Abstract

Selecting an appropriate statistical method is a challenge frequently encountered by applied researchers, especially if assumptions for classical, parametric approaches are violated. To provide some guidelines and support, we compared classical hypothesis tests with their typical distributional assumptions of normality and homoskedasticity with common and easily accessible alternative inference methods (HC3, HC4, and six bootstrap methods) in the framework of ordinary least squares (OLS) regression. The method’s performance was assessed for four different regression models with varying levels of non-normality and heteroskedasticity of errors, and for five different sample sizes ranging from 25 to 500 cases. For each scenario, 10,000 samples of observations were generated. Type I error and coverage rates, power, and standard error bias were examined to assess the methods’ performance. No method considered here performed satisfactorily on all accounts. Using HC3 or HC4 standard errors, or a wild bootstrap procedure with percentile confidence intervals, could yield reliable results in many, but not all, scenarios. We suppose that, in the case of assumption violations, researchers might refer to a method that performed best in a scenario most similar to their data situation. To aid the selection of an appropriate method, we provide tables comparing relative performances in all considered scenarios.

## Introduction

To this day, practical researchers in psychology or the social sciences are challenged with choosing the most suitable statistical method for their particular data situation. This is especially difficult if assumptions are not met when using well-established methods within the framework of the general linear model. Further, it has been shown that these assumptions are frequently violated in psychological research, sometimes even more often than they are met (Blanca et al., 2013; Bono et al., 2017; Micceri, 1989; Sladekova & Field, 2024b).

The general linear model family comprises many typical analyses, such as correlation analysis, linear regression, *t* tests or analyses of (co)variance. If the assumptions, on which the applicability of these analyses to psychological research questions is based, are not satisfied, results will be biased in some way, at times with drastic consequences as outlined further below.

Despite the availability of numerous alternatives to the general linear model that impose less restrictive assumptions (Wilcox, 2022), it often remains the framework of choice for statistical analysis (Blanca et al., 2018; Torres & Akbaritabar, 2024). The reason is likely that popular software packages, like SPSS, lack the necessary modules for all alternative analyses, coupled with a tendency of researchers to use models they are familiar with (Sladekova & Field, 2024c). Hence, it seems important to provide applied researchers with easily accessible alternative modes of inference that operate within the well-known realm of the general linear model while simultaneously avoiding issues associated with violations of assumptions.

### The starting point: Ordinary least squares regression

In this paper, we explore the performance of a few well-known alternative methods for some typical data scenarios. In particular, we focus on linear relationships between continuous variables typically assessed within the framework of the general linear model. One reason is that other relationships between variables can be easily expressed within the same general framework. For instance, a correlation can be expressed as a simple regression model with a continuous outcome and a continuous predictor. Similarly, an independent sample *t* test or between-subject factors ANOVA can be expressed as linear models with dummy coded predictors instead of continuous ones.

Since both correlation analyses as well as multiple predictor regression models are common in psychological research (Blanca et al., 2018), both are examined in this study. Thus, four different relationships between variables are specifically considered, which will hereinafter be referred to as “models”. The first model refers to two uncorrelated variables, one predictor and one outcome variable. The second model again refers to one predictor and one outcome, but this time, they are correlated with each other. The third model refers to two predictors and one outcome variable, whereas the latter only depends on one of the predictors but is independent of the other. The fourth model refers again to two predictors, this time both of which determine the value of the outcome variable.

In all scenarios, the predictors are known precisely, whereas the outcome is additively composed of its linear relationship with the predictor(s) and some unknown error. That means that the relationship between the predictor(s) and outcome variables can mathematically be described by the following equation:

1 $${y}_{i}=\sum\nolimits_{k=0}^{K}{\beta}_{k}{x}_{ik}+{\varepsilon}_{i}= {\beta}_{0}+{\beta}_{1}{x}_{i1}+\dots +{\beta}_{K}{x}_{iK}+{\varepsilon}_{i}$$

with $i=1,\dots ,n$ denoting the $i$-th out of overall $n$ observations (or cases), ${x}_{ik}$ the value of the $k$-th predictor for the $i$-th observation, and ${\varepsilon}_{i}$ denoting the error of the $i$-th observation. The parameters (also known as regression coefficients) ${\beta }_{k}$ with $k=0,\dots ,K$ express the linear relationship between the outcome ${y}_{i}$ and the predictors ${x}_{ik}$. For this reason, capital $K$ denotes the number of predictors in a model. If ${x}_{ik}$ increases by one unit (i.e., the value 1) while all other terms are kept constant at the right-hand side of Eq. (1), then the change of ${y}_{i}$ is given by ${\beta }_{k}$. For this reason, the parameters ${\beta}_{k}$ are sometimes also referred to as regression slopes, because they literally express how much the outcome increases/decreases if the respective predictor, and only that one, changes by one unit. The parameter ${\beta}_{0}$ is not linked to any predictor due to ${x}_{i0}:=1$ and is also referred to as the intercept. This name comes from the fact that ${\beta}_{0}$ equals the value at which the y-axis is intercepted by the regression line (in case of one predictor) or (hyper-)plane (in case of multiple predictors), making it the expected value of the outcome when all predictors are exactly zero.

Equation (1) can also be written in matrix notation:

2 $${\boldsymbol{y}}={\boldsymbol{X}}{\boldsymbol{\beta}}+{\boldsymbol{\varepsilon}}$$

where ${\boldsymbol{y}}$ and ${\boldsymbol{\varepsilon}}$ are vectors of length $n$ containing the values of the outcome variable and errors for all $n$ observations, $\beta$ is a vector of length $p$, with $p=K+1$, including the parameters ${\beta}_{k}$ with $k=0,\dots,K$ and ${\boldsymbol{X}}$ is an $n\times p$ matrix, including all the values ${x}_{ik}$ as in Eq. (1) with $i=1,\dots ,n$ (indicating the $i$-th row of the matrix) and $k=0,\dots,K$ (indicating the $\left(k+1\right)$-th or $p$-th column). Note that the first column of ${\boldsymbol{X}}$ is simply made up of $n$ ones due to ${x}_{i0}:=1$.

For any given real data set, that is, a set of $n$ observations of outcomes, ${y}_{i}$, and predictor variables, ${x}_{ik}$, the first task for an applied researcher then typically consists of estimating the parameters ${\beta}_{k}$ from that given data set. This is anything but straightforward, as evidenced by Eqs. (1) and (2), some portion of every observation ${y}_{i}$ is determined by some undefined error, meaning that we, in principle, do not know how erroneous an individual observation is. A typical way of dealing with this inevitable uncertainty is to search for an estimate of ${\boldsymbol{\beta}}$ that minimizes the contribution of the error term in Eqs. (1) and (2). The resulting solution should describe the outcome variable as much as possible by the supposed linear relationship with the predictors and as little as possible by the errors.

One such widely established solution became known as ordinary least squares (OLS) regression. In OLS regression, the ${\beta}_{k}$ are determined such that the sum of the squared residuals (RSS), i.e., the expression

3 $$\mathrm{RSS}= \sum\nolimits_{i=1}^{n}{r}_{i}^{2}=\sum\nolimits_{i=1}^{n}{({y}_{i}-{\widehat{y}}_{i})}^{2}=\sum\nolimits_{i=1}^{n}{({y}_{i}-{\sum }_{k=0}^{K}{\widehat{\beta }}_{k}{x}_{ik})}^{2}$$

becomes minimal. In Eq. (3), the ${r}_{i}$ with $i=1,\dots,n$ denote the so-called residuals, which are simply given by the difference between the observed values of the outcome variable ${y}_{i}$ and the predicted outcome values ${\widehat{y}}_{i}={\sum\nolimits }_{k=0}^{K}{\widehat{\beta}}_{k}{x}_{ik}$ with some chosen values for the parameters ${\widehat{\beta}}_{k}$.

Note that ${\widehat{\beta }}_{k}$ are not the true regression parameters ${\beta}_{k}$ but are instead those estimates of ${\beta }_{k},$ which minimize the sum of the squared residuals. Referring to the matrix representation introduced in Eq. (2), they are given by

4 $$\widehat{{\boldsymbol{\beta}}}={\left({{\boldsymbol{X}}}^{\mathrm{T}}{\boldsymbol{X}}\right)}^{-1}{{\boldsymbol{X}}}^{\mathrm{T}}{\boldsymbol{y}}$$

where $\widehat{{\boldsymbol{\beta}}}$ denotes the vector of length $p=K+1$ containing the estimated parameters ${\widehat{\beta }}_{k}$ with $k=0,\dots ,K$, ${{\boldsymbol{X}}}^{\mathrm{T}}$ denotes the transpose of the matrix ${\boldsymbol{X}}$, and ${\left({{\boldsymbol{X}}}^{\mathrm{T}}{\boldsymbol{X}}\right)}^{-1}$ denotes the inverse of the matrix ${{\boldsymbol{X}}}^{\mathrm{T}}{\boldsymbol{X}}$. Under specific conditions, the OLS estimate has some statistically favorable properties, such as representing the best linear unbiased estimator (BLUE) of the true regression coefficients. These conditions or assumptions are given further below and detailed descriptions can be found in Berry (1993).

### Classical inferential statistics in the framework of OLS regression

Especially important for the present work are conditions related to inferential statistical methods, that is, methods that allow us to quantify the uncertainty associated with the parameter estimates. For instance, say the OLS estimator yields a positive regression coefficient for the relation between a specific predictor and the outcome in our sample, how certain can we be that such a positive relation exists in the population? If the data fulfill certain requirements, these questions can be answered by classical hypothesis testing to some extent (Jones & Tukey, 2000). For instance, if the errors in Eqs. (1-2) can be construed as independent, normally distributed random variables with mean zero and constant variance, then the estimates of the parameters obtained by OLS also follow a normal distribution (Berry, 1993). Additionally, the spread of this theoretical normal distribution of estimated parameters is captured by an estimate of its standard deviation, known as the standard error (SE) of the parameter (Cohen et al., 2003). Computing a ratio of the estimated parameters and their standard errors results in a test statistic that follows a *t*-distribution. By comparing it with a critical value or computing a *p* value, classical null hypothesis significance testing (NHST) can be applied to arrive at a sensible decision about the sign of the true regression coefficient. However, the validity of this process and the reliability of its results stand and fall with the assumption that the data fulfill those specific conditions in the first place.

To be more precise, unbiased estimates of both regression parameters and their standard errors require that several assumptions about the data are met. These assumptions include error-free measurement of all predictors, errors with an expected value of zero and constant variance over the whole range of predictors (the latter is known as the homoskedasticity assumption), independence of errors, normally distributed errors, and various additional criteria depending on the source (Berry, 1993). Furthermore, all this rests on the condition that a linear relation exists between the predictors and the outcome, as expressed by Eqs. (1) and (2) truly exists up to an error term meeting these assumptions (Williams et al., 2013).

Some assumptions, like error-free measurement of the predictors, impact the estimate of the regression parameter itself. For instance, in a bivariate scenario having measurement error in the predictor will attenuate its effect on the outcome (Shear & Zumbo, 2013). However, this paper will focus only on two assumptions that do not impact the parameter estimation itself. Instead, the two assumptions considered impact only inferential methods following OLS regression in typical psychological applications (i.e., significance tests of regression parameters). The two assumptions are homoskedasticity and normality of the errors.

Note that in applications in the framework of hierarchical or time series data structures, the assumption about independence of errors is often violated, and alternative analyses (multilevel model, time series analysis, cluster robust SEs, etc.) should be considered (Williams et al., 2013). However, the assumptions about the distribution and the constant variance of the errors can often be managed within the familiar framework of the linear model simply by using adjusted standard errors or relying on bootstrap methods for significance testing (Astivia & Zumbo, 2019). Since both methods are readily available in popular software packages, we focus on these methods in this work and introduce them shortly in the following section.

### Heteroskedasticity-consistent standard errors

As outlined above, classical significance testing of regression parameters assumes a very specific error structure, especially that all errors have the same variance and do not covary with each other. In practice, the assumption of constant variance can, to some extent, be explored visually with the $K$ partial scatter plots between all predictors and the residuals of the model. Incorporating a regression line to each plot can at least give some impression of the spread of the residuals with respect to the predictors. If, for instance, it becomes apparent that the variability of the residuals around the regression line is different for different predictor levels, the assumption of constant error variance could be doubted. Using an inferential test based on that assumption in spite of this could result in a biased standard error, often leading to drastically inflated type I error rates (Cribari-Neto, 2004; Long & Ervin, 2000).

All different versions of the heteroskedasticity-consistent (HC) standard errors have been developed to account for this issue in OLS’s estimation of the standard error. They correct the standard errors of the regression coefficients by using information about the (difference in) variability of the residuals in the data. Two of the most widely used and recommended estimators are the HC3 and HC4 standard errors (Hayes & Cai, 2007). Both not only incorporate information about the residuals’ variability at different levels but also transform the residuals using the leverage values to accommodate their differing influences.

Previous studies have underlined the value of using either the HC3 or HC4 over conventional standard errors for inference when the heteroskedasticity assumption is not met. It had further been found that HC4 had superior finite-sample behavior and could outperform HC3 in samples with influential observations (Cribari-Neto, 2004; Hayes & Cai, 2007).

### Bootstrap procedures

Another approach to significance testing under less restrictive conditions is not to correct the standard errors, but instead to attempt to reproduce the sampling distribution of the parameter of interest empirically. The basic idea is to view a given sample of $n$ observations simply as their population and produce new samples of the same size by drawing observations from the given sample with replacement. Then, for each of these so-called bootstrap samples, the OLS estimate of regression parameters is computed. By drawing many bootstrap samples in this way, an empirical distribution of such estimates is generated. Typically, at least 1000 bootstrap samples are drawn to ensure a reasonably dense estimate of the sampling distribution.

Significance testing can be performed in various ways, including using the standard deviation of the bootstrap sampling distribution as an estimate of the standard error of the respective parameter, computing a bootstrap *p*-value, or constructing confidence intervals (CI) from the bootstrap sampling distribution (Hesterberg, 2011). SPSS, as well as a lot of other commercial software, allows for significance testing via two types of bootstrap confidence intervals, percentile and bias-corrected and accelerated (BCa), and a bootstrap *p* value. All of these approaches will be compared in this study.

The bootstrap approach described above is also known as the pairs bootstrap or simply case resampling, because it is the cases themselves that are being resampled. Here, a case denotes the combination of an observed outcome variable with the predictor values associated with it. In SPSS, the pairs bootstrap is called the “simple bootstrap” and is selected as the default.

However, the resampling itself can be performed in various ways (MacKinnon, 2006). For instance, one bootstrap method has been developed explicitly for a heteroskedastic error structure and is known as the wild bootstrap (MacKinnon, 2013). Its approach is first to compute the OLS estimate of regression parameters using the given sample. Then, the resulting residuals are saved, transformed, and multiplied by random noise before being used to compute the different bootstrap samples. This method is further elaborated on in the methods section.

While HC standard errors enable a conventional computation of test statistics and corresponding *p*-values, inference through bootstrap methods often relies on confidence intervals or *p* values derived from the bootstrap sampling distribution. Both bootstrap methods and methods based on adjusted standard errors have been recommended to resolve issues with inference in the framework of the general linear model if respective assumptions are violated (Astivia & Zumbo, 2019; Davidson & Flachaire, 2008).

### Specific objectives of the present work

The overall objective of this paper is to compare some of these well-established and easily accessible methods with the conventional approach for the four data scenarios introduced above. The methods will be compared regarding type I error rates, power, confidence interval (CI) coverage and standard error bias. The goal is to highlight the type of data situation for which a particular inferential method seems preferable and when it might still be appropriate to stick with the conventional approach. The latter is particularly interesting for violations of the distributional assumptions since many textbooks argue that classical significance testing of OLS estimators is mostly robust due to the central limit theorem (Pek et al., 2018). Prior simulation studies have reported that conventional inference generally works well when the normality assumption is violated, except when additionally extreme outliers are present (Knief & Forstmeier, 2021). Conversely, other authors caution against generalizing claims of robustness for conventional inferential methods when distributional assumptions are violated (Field & Wilcox, 2017).

For this reason, the data simulated for this study were generated using three error distributions (normal, moderately skewed, and highly skewed) and four degrees of error variability ranging from homoskedastic to highly heteroskedastic error structures. Additionally, the source of heteroskedastic errors in some data-generating models originates from the predictor of interest (first and second model), and in others from another predictor, not of primary interest to the researcher, like in the case of a control variable (third and fourth model). To our knowledge, the question of how heteroskedasticity introduced by one predictor may affect inference about parameters associated with another predictor has only rarely been addressed so far. Nonetheless, one previous simulation study had shown that the classical inference method primarily yielded inflated rejection rates for a given effect if heteroskedasticity was a function of the same predictor, but not if it was a function of a different predictor (Long & Ervin, 2000).

While this study does not explicitly examine the impact of outliers on performance measures, employing highly skewed error distributions often results in data with outliers present (Knief & Forstmeier, 2021).

## Methods

### Data simulation

To examine a diverse range of data situations, four different data-generating models, three error distributions, four levels of heteroskedasticity, and five sample sizes were simulated. All conditions are summarized in Table 1.

**Table 1:** Summary of simulated conditions

Parameters	Values
Models	${y}_{{\boldsymbol{i}}}=0+0\cdot {x}_{i1}+{\varepsilon}_{i}$, ${y}_{{\boldsymbol{i}}}=0+0.8\cdot {x}_{i1}+{\varepsilon}_{i}$, ${y}_{{\boldsymbol{i}}}=0+0\cdot {x}_{i1}+0.3\cdot {x}_{i2}+{\varepsilon}_{i}$, ${y}_{{\boldsymbol{i}}}=0+0.8\cdot {x}_{i1}+0.3\cdot {x}_{i2}+{\varepsilon}_{i}$
Sample size	25, 50, 100, 200, 500
Distribution	Normal (skewness = 0, kurtosis = 0), Moderately non-normal (skewness = 2, kurtosis = 7), Severely non-normal (skewness = 3, kurtosis = 21),
Heteroskedasticity levels (g)	Homoskedastic ($g=0$), Slightly heteroskedastic ($g=0.5$), Moderately heteroskedastic ($g=1$), Severely heteroskedastic ($g=2$)

The conditions “sample size = 25” and “distribution = severely non-normal” were not paired because of the constraint to achieve a sample representative of the population from which samples were drawn, resulting in the following pairings of sample size and distribution conditions: 4 (sample size) × 3 (distributions) + 1 (sample size) × 2 (distributions) = 14. Therefore, the total number of simulated conditions was 14 (distributions × sample sizes) × 4 (models) × 4 (heteroskedasticity levels) = 224

Two one-predictor regression models and two two-predictor regression models were considered for the linear relationship between predictors ${x}_{ik}$ and outcome ${y}_{i}$, each of which either included an effect of interest (denoted as ${x}_{i1}$) of zero, to investigate type I error, or included an effect of interest (denoted as ${x}_{i1}$) of 0.8, to explore power. This large true effect of 0.8 was chosen for models 2 and 4 so that a reduction in power due to a change in method or change in scenario is reflected in the results in high resolution. The one-predictor models represent correlational analyses common in psychological research (Blanca et al., 2018), and the two-predictor models allow for variation in the source of heteroskedasticity, which has also been shown to impact rejection rates (Long & Ervin, 2000).

The index $i$ ranges from 1 to $n$ with $n$ denoting one of the five possible sample sizes (i.e., 25, 50, 100, 200, or 500). Each modeled linear relationship was combined with an error term according to Eqs. (1) and (2) and modeled as outlined above. Finally, specific (true) regression weights were chosen for the predictors as follows:

- Model 1:
  5 $${y}_{{\boldsymbol{i}}}=0+0\cdot {x}_{i1}+{\varepsilon}_{i},$$
- Model 2:
  6 $${y}_{{\boldsymbol{i}}}=0+0.8\cdot {x}_{i1}+{\varepsilon}_{i},$$
- Model 3
  7 $${y}_{{\boldsymbol{i}}}=0+0\cdot {x}_{i1}+0.3\cdot {x}_{i2}+{\varepsilon}_{i,}$$
- Model 4:
  8 $${y}_{{\boldsymbol{i}}}=0+0.8\cdot {x}_{i1}+0.3\cdot {x}_{i2}+{\varepsilon}_{i}$$

In Eqs. (5)-(8), ${\varepsilon }_{i}$ denotes the simulated error for the $i$-th observation, and $i=1,\dots,n$ with $n\in \left\{25, 50, 100, 200, 500\right\}$.

The predictors, in each of the four models, were drawn from a normal distribution with mean 0 and variance 1. The distribution of the predictor variables is inconsequential for the classical inference method in OLS regression (Williams et al., 2013), so a normal distribution was chosen simply because many researchers are familiar with it. The mean of 0 and the variance of 1 make the variables well-known *z*-standardized variables, that any (continuous) variable in any data set can be transformed to. The predictors were chosen to have a correlation of $r= .10$, because researchers rarely have perfectly uncorrelated predictors in practice. Based on conventions the chosen correlation would be categorized as a small effect (Cohen, 1988). However, this choice is completely arbitrary and could also be systematically varied in subsequent simulations (see e.g. Long & Ervin, 2000).

As mentioned above, simulated data sets were of sizes 25, 50, 100, 200, and 500, to evaluate performance in a range of sample sizes realistically encountered by applied researchers.

Additionally, for each of the four models with five different sample sizes each, different combinations of error distributions and heteroskedasticity were generated. The error of the *i*-th observation was computed as ${\varepsilon}_{i}={u}_{i}\ast {\sigma}_{i}$. The ${u}_{i}$, with a mean of 0 and a variance of 1, were drawn from three different distributions of increasing non-normality: Normal (skew = 0, kurtosis = 0), moderately non-normal (skewness = 2, kurtosis = 7) and severely non-normal (skewness = 3, kurtosis = 21). The values were chosen based on prior simulation studies (Curran et al., 1996; Nevitt & Hancock, 2001). In this paper, kurtosis always refers to the excess kurtosis, with excess kurtosis = kurtosis – 3, so that a normal distribution is defined to have a kurtosis of zero. The function *fleishman_sim* (based on Fleishman, 1978) from the R package *miceadds* (version 3.17-44) was used to generate the random variable $u$ from a univariate distribution with a specific skew and kurtosis (Robitzsch & Grund, 2024). However, for the smallest sample size of 25 cases, it was not possible to reliably draw samples that matched the population’s severely non-normal shape, which is why this condition could only be generated for sample sizes of 50 and larger. Additionally, since the skewness and kurtosis values of the empirical distribution can differ substantially from the values of the population they are drawn from, bounds were put on the empirical skewness and kurtosis values in the simulation. Specifically, vectors of ${u}_{i}$ of a given sample size were drawn until their empirical distribution’s skewness and kurtosis values both were reasonably close to the chosen values, for each round of simulating a single data set. Reasonably close meant that the empirical values were between – 1 and 1 (skewness and kurtosis) for the normal condition, 1 and 3 (skewness) and 6 and 8 (kurtosis) for the moderately non-normal condition, and 2 and 4 (skewness) and 19 and 23 (kurtosis) for the severely non-normal condition.

Restricting the sampling process with regard to the distributional form of the sampled errors was done to

- Counteract a downward bias in skewness and kurtosis and
- Reflect that often alternative analyses are only considered if the sample residual distribution exhibits noticeable non-normal features.

Still, 1000 replications without constraining sampling were also examined. Results were comparable regardless of the sampling procedure. This is further expanded on in the appendix. Therefore, we continue to refer to the proportion of significant results as type I error and power, for a zero and 0.8 true effect, respectively, with the caveat that the sampling process was not strictly random.

To include different sources of heteroskedasticity, ${\sigma}_{i}$ was computed as ${\sigma}_{i}^{2}={e}^{g\ast{x}_{i1}}$ for models 1 and 2 in Eqs. (5) and (6), and as ${\sigma}_{i}^{2}={e}^{g\ast{x}_{i2}}$ for models 3 and 4 in Eqs. (7) and (8). The values of $g$ characterize the four heteroskedasticity levels: $g=0$, $g=0.5$, $g=1$, $g=2$. A $g$ of zero results in no heteroskedasticity, because every $i$-th observation has the same error variance ${\sigma}_{i}^{2}$ (and standard deviation ${\sigma}_{i}$) of 1. Increasing $g$ results in error variances that differ between the $n$ observations, thus inducing heteroskedasticity as a function of either ${x}_{1}$ or ${x}_{2}$.

Overall, this resulted in 56 (60 minus 4) scenarios for each of the modeled linear relationships expressed by Eqs. (5) to (8) due to five possible sample sizes, three considered error distributions, four considered levels of heteroskedasticity, and the fact that for the smallest sample size of 25 cases, it was not possible to reliably draw samples that matched the population’s severely non-normal shape. Ten thousand replications were generated for each scenario. All calculations and analyses outlined above were performed in R (version: 4.3.3) and RStudio.

### Method performance

For each generated replication, an OLS regression was performed, followed by the computation of classical, HC3 and HC4 standard errors for statistical inference about the regression parameters. In addition, two bootstrap sampling methods (pairs and wild bootstrap) were used for inference via the SPSS version of bootstrap *p* values, percentile confidence intervals and bias-corrected and accelerated (BCa) confidence intervals. In total, this resulted in nine different methods. Significance levels were chosen as $\alpha =.05$ in each case, meaning that results with a *p*-value <.05 in a two-sided significance test or results where the 95% confidence interval did not include zero were deemed significant. All methods used for inference are described in detail in the following.

Each method’s performance was assessed with type I error rates for model 1 and 3 (Eqs. 5, 7), and power and coverage rates for model 2 and 4 (Eqs. 6, 8).

Type I error rate and coverage rate are closely related and often yield identical results in the special case where the true regression parameter is zero, given that they share the same nominal alpha value. The reason is that in the long run, under repeated sampling, the confidence interval is expected not to cover the true parameter of zero in exactly a proportion alpha of all cases. Equivalently, under the same conditions, the null-hypothesis test is expected to falsely reject the null-hypothesis that the true regression parameter is zero with a probability of alpha.

Statistical power is understood as the probability of rejecting a null-hypothesis with NHST, given the null-hypothesis is false (Cohen, 1988). It can also be expressed as the complementary probability of the type II error. Thus, power is related to the type I error in the sense that setting a more stringent, smaller alpha value for a null-hypothesis test will lead to a reduction in power, if the size of the true effect and the sample size remain unchanged. For a constant nominal alpha level, increasing the sample size or the effect of interest, will lead to an increase in power. Typically, methods that show rejection rates greater than the nominal alpha value, would be expected to have higher power and vice versa. Similarly, methods with a very high power might suffer from inadequate coverage of the true parameter, due to confidence interval bounds that are too narrow.

In models 1 and 3, where the true effect was zero, a method of inference works as intended if the proportion of significant results out of all 10,000 repetitions equals approximately .05. That is, if it rejects the hypothesis that the regression parameter equals zero approximately as often as indicated by the nominal significance value $\alpha$, that is, in about 5% of all cases.

Since only a finite number of replications can be simulated, slight deviations from the nominal alpha level are to be expected. However, at which point deviations are no longer classified as negligible, should be clearly defined. Bradley (1978) proposed two criteria: a stringent criterion where the method is regarded as robust if the proportion of significant results lies between 0.9*α and 1.1*α, and a liberal criterion with a range of 0.5*α to 1.5*α. With $\alpha =.05$ like in the present work, the stringent criterion ranges from .045 to .055 and the liberal criterion ranges from .025 to .075. In the results section, methods will be classified as being within either the stringent or liberal criterion, being overly conservative, i.e., having rejection rates below 2.5%, or being overly liberal, i.e., having rejection rates above 7.5%.

In models 2 and 4, where the true effect was 0.8, analogous criteria as for the type I error rates are used for the coverage rates: The stringent criterion lies within .945 and .955, and the liberal criterion lies within .925 and .975. Coverage rates smaller than .925 are too liberal as the confidence intervals are too narrow, and coverage rates larger than .975 are too conservative, as confidence intervals are too broad.

Methods achieving a power of under 80% will be categorized as underpowered in the results section. As a convention, a minimum power of.80 (type II error =.20) for classic null-hypothesis-testing has been widely adopted (Cohen, 1988). For an alpha level of.05 this would set the type I error to be four times as serious as the type II error. A different power criterion is that the type II error should be seen as equally serious as the type I error, in which case a power of.95 would be desirable (Cohen, 1988).

### Robust methods computation

In the Introduction, section Eq. (4) showed the computation of the vector $\widehat{{\boldsymbol{\beta}}}$ that contains all estimated regression coefficients ${\widehat{\beta}}_{k}$, with $k=0, \dots, K$. In order to do classical null-hypothesis testing on these regression coefficients, each parameter’s variance has to be estimated. Taking the square root of the estimated parameter’s variance results in its respective standard error. In classical inference typical for OLS regression, the variance of $\widehat{{\boldsymbol{\beta}}}$ is computed as follows:

9 $$Var\left(\widehat{{\boldsymbol{\beta}}}\right)=\widehat{{\boldsymbol{\Phi}}}={({{\boldsymbol{X}}}^{\mathrm{T}}{\boldsymbol{X}})}^{-1}{{\boldsymbol{X}}}^{\mathrm{T}}\widehat{{\boldsymbol{\Omega}}}{\boldsymbol{X}}{({{\boldsymbol{X}}}^{\mathrm{T}}{\boldsymbol{X}})}^{-1},$$

where $\widehat{{\boldsymbol{\Omega}}}$ is an $n\times n$ matrix with ${\widehat{\sigma}}^{2}$ in the diagonal and zeros in the off-diagonal elements. ${\widehat{\sigma }}^{2}$ is an estimator for the variance of the errors ($Var\left(\varepsilon \right)={\sigma}^{2}$) and is computed as the sum of the squared residuals (RSS) from Eq. (3) divided by its degrees of freedom $n-p$. When assuming a constant error variance, Eq. (9) can be simplified to $Var\left(\widehat{{\boldsymbol{\beta}}}\right)={\widehat{\sigma}}^{2}{({{\boldsymbol{X}}}^{\mathrm{T}}{\boldsymbol{X}})}^{-1}$.

### Heteroskedasticity-consistent standard error

However, if the true variance structure of the errors more closely resembles a covariance matrix, where each error has a different variance, such as:

10 $$Var\left(\varepsilon \right)=\left[\begin{array}{cccc}{\sigma}_{1}^{2}& 0& \dots & 0\\ 0& {\sigma}_{2}^{2}& \dots & 0\\ \vdots & \vdots & \ddots & \vdots \\ 0& 0& \dots & {\sigma}_{n}^{2}\end{array}\right]={\boldsymbol{\Omega}},$$

estimating a single error variance parameter ${\widehat{\sigma }}^{2}$ would not be justified.

To avoid bias in the variance of $\widehat{{\boldsymbol{\beta}}}$ in these instances, a variety of so-called sandwich estimators have been proposed. Because of their superior performance in prior research (Hayes & Cai, 2007), HC3 and HC4 are chosen for this study.

Both HC3 and HC4’s estimates of the variance of $\widehat{{\boldsymbol{\beta}}}$ look very similar to the classical standard error estimator. They just replace $\widehat{{\boldsymbol{\Omega}}}$ with a different matrix, where the elements on the diagonal are the squared $i$-th residuals weighted by the $i$-th leverage value (${h}_{ii}$). The structure of both HC estimators is nearly identical, with the only difference being the exponent of the expression in the denominator, with:

11 $$HC3={({{\boldsymbol{X}}}^{\mathrm{T}}{\boldsymbol{X}})}^{-1}{{\boldsymbol{X}}}^{\mathrm{T}}diag\left[\frac{{r}_{i}^{2}}{{(1-{h}_{ii})}^{2}}\right]{\boldsymbol{X}}{({{\boldsymbol{X}}}^{\mathrm{T}}{\boldsymbol{X}})}^{-1},$$

and

12 $$HC4={({{\boldsymbol{X}}}^{\mathrm{T}}{\boldsymbol{X}})}^{-1}{{\boldsymbol{X}}}^{\mathrm{T}}diag\left[\frac{{r}_{i}^{2}}{{(1-{h}_{ii})}^{{\delta }_{i}}}\right]{\boldsymbol{X}}{({{\boldsymbol{X}}}^{\mathrm{T}}{\boldsymbol{X}})}^{-1},$$

where

13 $${\delta }_{i}=min\left\{4, \frac{n{h}_{ii}}{p}\right\}$$

HC3 simply squares the expression in the denominator, whereas HC4 adds emphasis on high leverage values by making the exponent dependent on them.

#### Bootstrap procedures

As described in the introduction, the pairs bootstrap takes $B$ bootstrap samples by randomly drawing $n$ cases, with replacement, from the original sample. For each iteration, a regression coefficient is computed using the data from the bootstrap sample, resulting in a distribution of bootstrap regression coefficients. In contrast, the wild bootstrap procedure is done in multiple steps:

- Compute a regression model with the original data set and save the residuals ($r$).
- Multiply the residuals by some random variable ${v}^{\ast}$ with mean 0 and variance 1, that is redrawn in each of the $B$ bootstrap iterations. Optionally, prior to this, the residuals are transformed by some predefined function (see below).
- Compute a new, bootstrap outcome variable ${y}^{\ast}$ in the $b$-th bootstrap iteration.
- Regress the $b$-th bootstrap outcome variable onto the set of original predictor variables and estimate the regression parameters ${\widehat{\beta }}_{b}^{\ast}$.

Even though the concept of the wild bootstrap stays the same, slightly different variations on this method exist. To enhance the practical utility of this study’s results for applied researchers, the bootstrap methods and inferences were recreated using the SPSS 29 algorithm manual (IBM, 2022). For the wild bootstrap, the transformation of the unstandardized residuals saved from the original regression model is often chosen to mirror the HC approaches for better performance (Flachaire, 2005; MacKinnon, 2013). The $i$-th residual is divided by $\sqrt{(1-{h}_{ii})}$ or $(1- {h}_{ii})$, depending on whether it should resemble HC2 or HC3, respectively. In SPSS the unstandardized deleted residuals can be saved which equals the HC3 approach. The transformed residual is then multiplied by some random variable ${v}^{\ast}$ with mean 0 and variance 1. In SPSS ${v}^{\ast}$ is drawn from a two-point distribution.

The wild bootstrap used in SPSS 29, and thus also in this paper, uses the following formula for steps b) and c) as described above to compute the $i$-th bootstrap outcome variable ${y}_{i}^{\ast}$:

14 $${y}_{i}^{\ast}={{\boldsymbol{X}}}_{i}\widehat{{\boldsymbol{\beta}}}+\frac{{r}_{i}}{(1-{h}_{ii})}{v}_{i}^{\ast},$$

where ${r}_{i}$ is the $i$-th residual from the original regression model (step a)), ${h}_{ii}$ is the $i$-th leverage value, ${{\boldsymbol{X}}}_{i}$ is the $i$-th row of the $n\times p$ matrix ${\boldsymbol{X}}$ that contains the original predictor values of the $i$-th case and $\widehat{{\boldsymbol{\beta}}}$ is the $p\times 1$ vector of estimated regression coefficients from step a). The random variable ${v}_{i}^{\ast}$ is drawn from the so-called Rademacher distribution,

15 $$F: {v}_{i}^{\ast}=\left\{\begin{array}{cc}1& \text{with probability}\,0.5,\\ -1& \text{with probability }0.5.\end{array}\right.$$

This means, ${v}_{i}^{\ast}$ can take on the values 1 or −1, essentially just keeping or changing the sign of the (transformed) residual. In step d) the bootstrap regression coefficients of the $b$-th bootstrap samples are then computed via OLS, that is, using ${\widehat{{\boldsymbol{\beta}}}}_{{\boldsymbol{b}}}^{\boldsymbol{\ast}}={\left({{\boldsymbol{X}}}^{\mathrm{T}}{\boldsymbol{X}}\right)}^{-1}{{\boldsymbol{X}}}^{\mathrm{T}}{{\boldsymbol{y}}}^{\boldsymbol{\ast}}$.

Once the $B$ bootstrap regression coefficients are obtained, different methods can be used to perform the null-hypothesis test. Two confidence interval methods and the bootstrap *p*-value, reported by SPSS, are compared here. The bootstrap *p*-value of the $k$-th predictor was computed as follows:

16 $${p}_{k}= \frac{\left|\left\{{{t}_{bk}^{\ast}}^{2}\ge {t}_{k}^{2}\right\}\right| + 1}{B+1}$$

with

17 $${t}_{b}^{\ast}=\frac{{\beta }_{bk}^{\ast}-{\widehat{\beta }}_{k}}{{SE}_{bk}^{\ast}}.$$

Here, ${\widehat{\beta }}_{k}$ is the $k$-th estimated regression coefficient of the regression model in the original sample and ${t}_{k}$ is its respective test statistic, commonly referred to as “t-value”. ${\widehat{\beta}}_{bk}^{\ast}$ is the $k$-th estimated regression coefficient in the $b$-th bootstrap sample, and $B$ is the number of bootstrap samples drawn. The term $\left|\left\{{{t}_{bk}^{\ast}}^{2}\ge {t}_{k}^{2}\right\}\right|$ denotes the count of all ${{t}_{bk}^{\ast}}^{2}$ that fulfill the condition $\left\{{{t}_{bk}^{\ast}}^{2}\ge {t}_{k}^{2}\right\}$. This study used a $B$ of 1,000 bootstrap samples. ${SE}_{bk}^{\ast}$ is the bootstrap standard error of the $k$-th regression coefficient in the $b$-th bootstrap sample:

18 $${SE}_{bk}^{\ast}=\sqrt{\frac{1}{B-1}{\sum }_{b=1}^{B}({\widehat{\beta}}_{bk}^{\ast}-{\overline{\beta}}_{k}^{\ast})},$$

where ${\overline{\beta}}_{k}^{\ast}$ denotes the mean of all $B$ bootstrap regression coefficients of the $k$-th predictor (${\widehat{\beta }}_{bk}^{\ast}$).

The two confidence intervals are referred to as the percentile and the bias-corrected and accelerated (BCa) confidence interval. The percentile bootstrap confidence interval simply uses the bootstrap sampling distribution of the bootstrap regression coefficients to compute the upper and lower bounds of the confidence interval. The upper bound is the value of ${\widehat{\beta }}_{b}^{\ast}$ beneath which 2.5% of values lie and the lower bound is the value of ${\widehat{\beta }}_{b}^{\ast}$ above which 97.5% of values lie. The BCa confidence interval takes the shape of the bootstrap sampling distribution into account. It contains a parameter to correct for bias in the estimate, along with an acceleration parameter to correct for skew in the distribution. In the past, using the BCa confidence interval has been reported to lead to increased power in some instances as well as increased type I error, specifically when testing the indirect effect in mediation analysis (Fritz et al., 2012). The exact formulas used in this paper to compute the BCa confidence intervals can be found in the appendix.

## Results

### Type I error

**Fig. 1:**
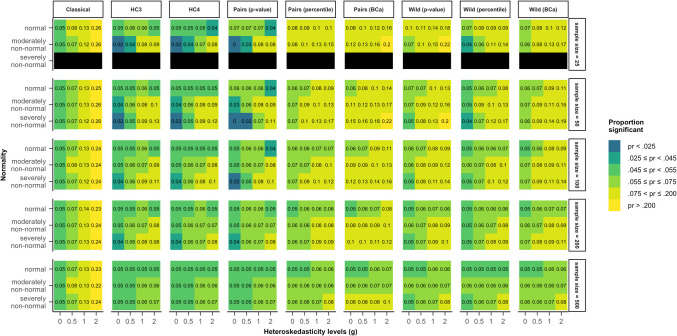
Type I error rates for all tested methods and data scenarios, with color-coded highlights depending on the deviation from the nominal alpha value (5%) in model 1. *Note*. The simulated data was modeled after Eq. (5) (model 1), where heteroskedasticity was generated as a function of the predictor of interest (${x}_{1}$). *Classical*: classical inference typical for OLS regression, *HC3* & *HC4*: inference based on versions of the heteroskedasticity-consistent standard errors (Eqs. 11, 12), *Pairs*: pairs bootstrap resampling method, *Wild*: wild bootstrap resampling method (Eq. 14), *p value*: inference based on the bootstrap *p* value (Eq. 16), *percentile*: inference based on the bootstrap percentile confidence interval, *BCa*: inference based on the bias-corrected and accelerated bootstrap confidence interval

Figure 1 shows the results of the nine different methods of inference on data generated by the first model given in Eq. (5), where heteroskedasticity was a function of the predictor of interest. In this case, the classical method consistently rejected the null-hypothesis too often in heteroskedastic samples. In particular, the type I error rate was inflated, with rejection rates up to 26% for the strongest heteroskedasticity condition. This result was essentially independent of the sample size and the distribution of the errors.

Both heteroskedasticity-consistent standard errors performed considerably better than the classical method, especially with increasing sample size. With normally distributed errors, their rejection rates stayed within the liberal criterion (2.5−7.5%) for all examined sample sizes, even under severe heteroskedasticity. In samples of 25 or 50 cases, both HC methods were too conservative when errors were homoskedastic but non-normal. On the contrary, in data situations with non-normal errors and moderate to severe heteroskedasticity HC3 and HC4 yielded type I error rates around 10% (highlighted by the yellow color in Fig. 1). Only in sample sizes of 500 did both methods stay within the liberal criterion for all combinations of heteroskedasticity and non-normality.

The bootstrap methods showed a greater variety in their performance in model 1 (Eq. 5). The pairs bootstrap using a bootstrap *p* value yielded similar results to HC3 and HC4, where heteroskedasticity in combination with non-normality led to rejection rates greater than 7.5%, but homoskedasticity in combination with non-normality led to rejection rates smaller than 2.5%. Both bootstrap confidence interval methods with the pairs bootstrap and the wild bootstrap with the bootstrap *p* value were too liberal in most scenarios, for instance even when both assumptions were met when the sample size was 25. The wild bootstrap with the percentile CI and the BCa CI showed rejection rates greater than 7.5% as soon as the errors were slightly heteroskedastic but fell within the liberal criterion when the errors were homoskedastic, independent of their distribution. Overall, the pairs bootstrap using a BCa confidence interval for inference yielded inflated type I error rates over the widest range of scenarios. Note, however, that no method performed satisfactorily in all scenarios, although all methods except for the classical inference method, showed rejection rates closer to 5% in many scenarios the larger the sample size.

**Fig. 2:**
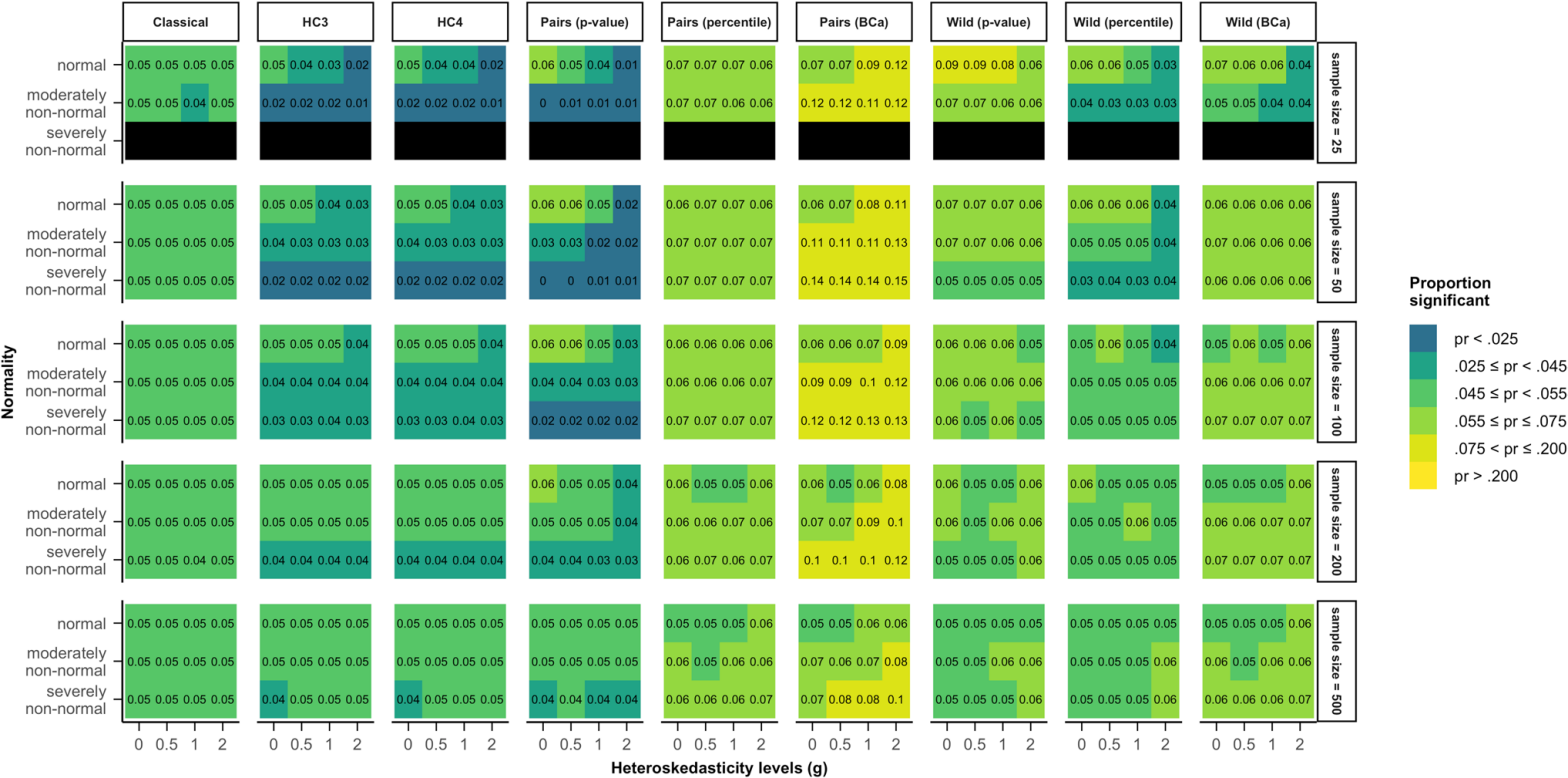
Type I error rates for all tested methods and data scenarios with color-coded highlights depending on the deviation from the nominal alpha value (5%) in model 3. *Note*. The simulated data was modeled after Eq. (7) (model 3), where heteroskedasticity was generated as a function of a different predictor (${x}_{2}$) not the predictor of interest (${x}_{1}$). *Classical*: classical inference typical for OLS regression, *HC3* & *HC4*: inference based on versions of the heteroskedasticity-consistent standard errors (Eqs. 11, 12), *Pairs*: pairs bootstrap resampling method, *Wild*: wild bootstrap resampling method (Eq. 14), *p value*: inference based on the bootstrap *p* value (Eq. 16), *percentile*: inference based on the bootstrap percentile confidence interval, *BCa*: inference based on the bias-corrected and accelerated bootstrap confidence interval

When the heteroskedasticity was generated as a function of another (correlated) predictor (${x}_{2}$) and not the predictor of interest (${x}_{1}$), that is, in the case of model 3, according to Eq. (7), type I error rates for the predictor of interest showed a different pattern for varying non-normality and heteroskedasticity of errors and sample sizes. In that case, the classical inferential method outperformed all other estimation methods and almost always kept rejection rates within the stringent criterion (4.5–5.5%), as shown by Fig. 2. Both HC standard errors were consistently conservative in samples of 50 cases or smaller, specifically for non-normal or heteroskedastic data. In larger sample sizes, their rejection rates fell within 2.5 and 5% for all scenarios, with values closer to 5% with increasing sample size.

Once more, the different bootstrap methods showed a larger variety. The pairs bootstrap with inference based on the bootstrap *p* value, yielded type I error rates similar to those of HC3 and HC4. However, the method was still overly conservative for samples of 100 cases, when the errors were severely non-normally distributed.

The pairs bootstrap using a percentile confidence interval yielded rejection rates between 4.5% and 7.5% across all scenarios. In contrast, the pairs bootstrap using a BCa confidence interval was overly liberal (rejection rates greater than 7.5%) in the majority of simulated scenarios. Even in the largest sample size of 500, its rejection rates reached 10% for heteroskedastic errors drawn from a severely non-normal distribution.

The wild bootstrap with the bootstrap *p* value performed adequately for samples of 50 cases and above, showing rejection rates between 4.5% and 7.5%. In small samples of 25, it was overly liberal for normally distributed errors. The wild bootstrap with the percentile CI and with the BCa CI kept type I error rates within the bounds of the liberal criterion (2.5–7.5%) in all scenarios.

Out of all the robust methods, the wild bootstrap with the percentile CI yielded rejection rates between 4.5% and 5.5% for the largest variety of scenarios, although all of them were outperformed by the classical inference method.

### Confidence interval coverage

As described in the methods section, coverage rate results are only reported for models with a true parameter different from zero (models 2 and 4). For the two methods, the pairs bootstrap with the *p* value and the wild bootstrap with the *p* value, coverage rates could not be reported because neither inference method allowed for the computation of a confidence interval.

Overall, the pattern of coverage rates in models 2 and 4 for specific scenarios that covered the true parameter too often or too little was very similar to the pattern of the type I error rates in models 1 and 3, respectively.

**Fig. 3:**
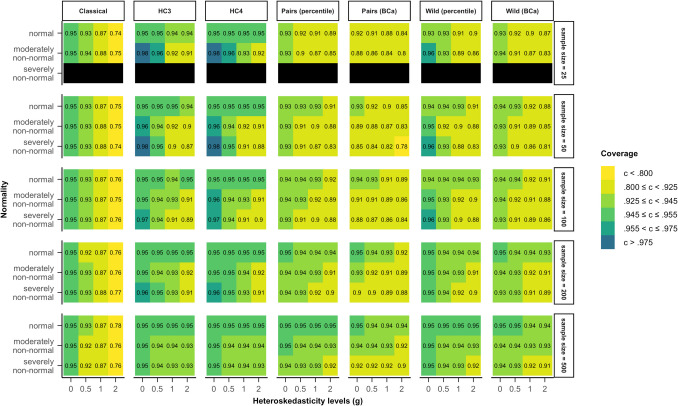
Coverage rates for all tested methods and data scenarios with color-coded highlights depending on the deviation from the nominal value (95%) in model 2. *Note*. The simulated data was modeled after Eq. (6) (model 2), where heteroskedasticity was generated as a function of the predictor of interest (${x}_{1}$). *Classical*: classical inference typical for OLS regression, *HC3* & *HC4*: inference based on versions of the heteroskedasticity-consistent standard errors (Eqs. 11, 12), *Pairs*: pairs bootstrap resampling method, *Wild*: wild bootstrap resampling method (Eq. 14), *percentile*: inference based on the bootstrap percentile confidence interval, *BCa*: inference based on the bias-corrected and accelerated bootstrap confidence interval

In Fig. 3, coverage rates for model 2 (Eq. 6), where heteroskedasticity was a function of the predictor of interest, are presented. For the classical inference method, an increase in heteroskedasticity led to a drop in coverage rate from the nominal 95% to approximately 75%, no matter the sample size. This means that when errors were severely heteroskedastic, the true regression coefficient was only within the bounds of the confidence interval in 75% of cases.

HC3 and HC4 showed different coverage rates depending on which assumption was violated. Under homoskedasticity with non-normally distributed errors, the coverage rate was larger than 97.5% in small samples, indicating that the bounds of the confidence intervals were too broad. Contrarily, with non-normally distributed errors that were also heteroskedastic, the confidence intervals were too narrow, resulting in coverage rates smaller than 92.5%. Generally, the coverage rates got closer to 95% with increasing sample size.

The two pairs bootstrap confidence intervals were generally too narrow and showed coverage rates smaller than 92.5% in a variety of scenarios where both the homoskedasticity and the normality assumption were not met. The bounds of the BCa confidence interval covered the true regression coefficient even less often than the percentile confidence interval. For the wild bootstrap, both the percentile and the BCa confidence intervals also yielded coverage rates that were too low if assumptions were not met. Out of all the bootstrap methods, coverage rates of the wild bootstrap with the percentile confidence interval lay within the bounds of the liberal criterion for the largest number of scenarios. Further, all bootstrap methods showed coverage rates closer to the nominal value with an increase in sample size.

**Fig. 4:**
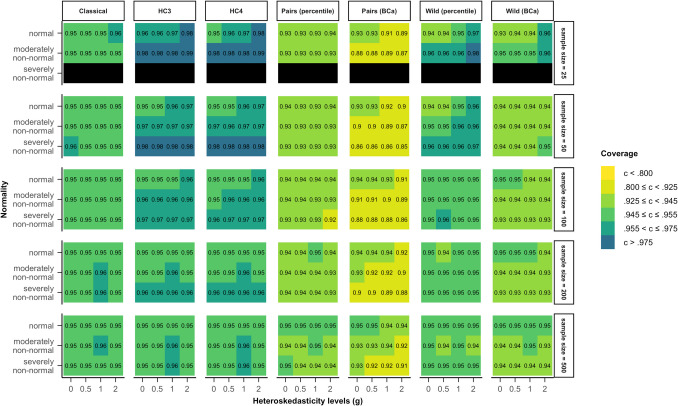
Coverage rates for all tested methods and data scenarios with color-coded highlights depending on the deviation from the nominal value (95%) in model 4. *Note*. The simulated data was modeled after Eq. (8) (model 4), where heteroskedasticity was generated as a function of a different predictor (${x}_{2}$) not the predictor of interest (${x}_{1}$). *Classical*: classical inference typical for OLS regression, *HC3* & *HC4*: inference based on versions of the heteroskedasticity-consistent standard errors (Eqs. 11, 12), *Pairs*: pairs bootstrap resampling method, *Wild*: wild bootstrap resampling method (Eq. (14)), *percentile*: inference based on the bootstrap percentile confidence interval, *BCa*: inference based on the bias-corrected and accelerated bootstrap confidence interval

Figure 4 presents the coverage rates of model 4 (Eq. 8), where heteroskedasticity was a function of a different predictor and not the predictor of interest. Once more, the pattern of deviations from the nominal coverage rate of 95% generally mirrored the pattern of deviations from the nominal alpha value in Fig. 2.

Out of all the examined methods, the classical inference method yielded coverage rate results within 94.5% and 95.5% for the greatest variety of scenarios. The confidence intervals based on the two HC standard error methods were generally too broad in small samples when errors were not normally distributed. In samples of 100 cases or more, their coverage rates lay between 94.5% and 97.5%.

The pairs bootstrap with the percentile confidence interval showed coverage rates between 92.5% and 95.5% in the majority of scenarios, no matter whether assumptions were violated or not. Combined with a BCa confidence interval, the pairs bootstrap yielded coverage rates that were lower than 92.5% in many scenarios, again signifying that the BCa confidence intervals were too narrow. The wild bootstrap yielded better results with both confidence interval methods. The coverage rate based on the BCa confidence interval stayed within 92.5% and 97.5%. The percentile confidence interval showed more scenarios were the coverage rate fell within the stringent criterion (94.5–95.5%), although it was slightly too conservative for the smallest sample size of 25 cases.

### Power

When generating a model, with a rather large population effect according to Eqs. (6) and (8), the rejection rate, i.e., the proportion of results flagged as significant by a method, can be seen as a power estimate for the respective method in each scenario.

For models 2 and 4 simulated in this study the classical inference method showed a power of 94% to detect the effect of interest, when all assumptions were met, and the sample size was 25. For all larger samples, the power was 100% in this scenario.

**Fig. 5:**
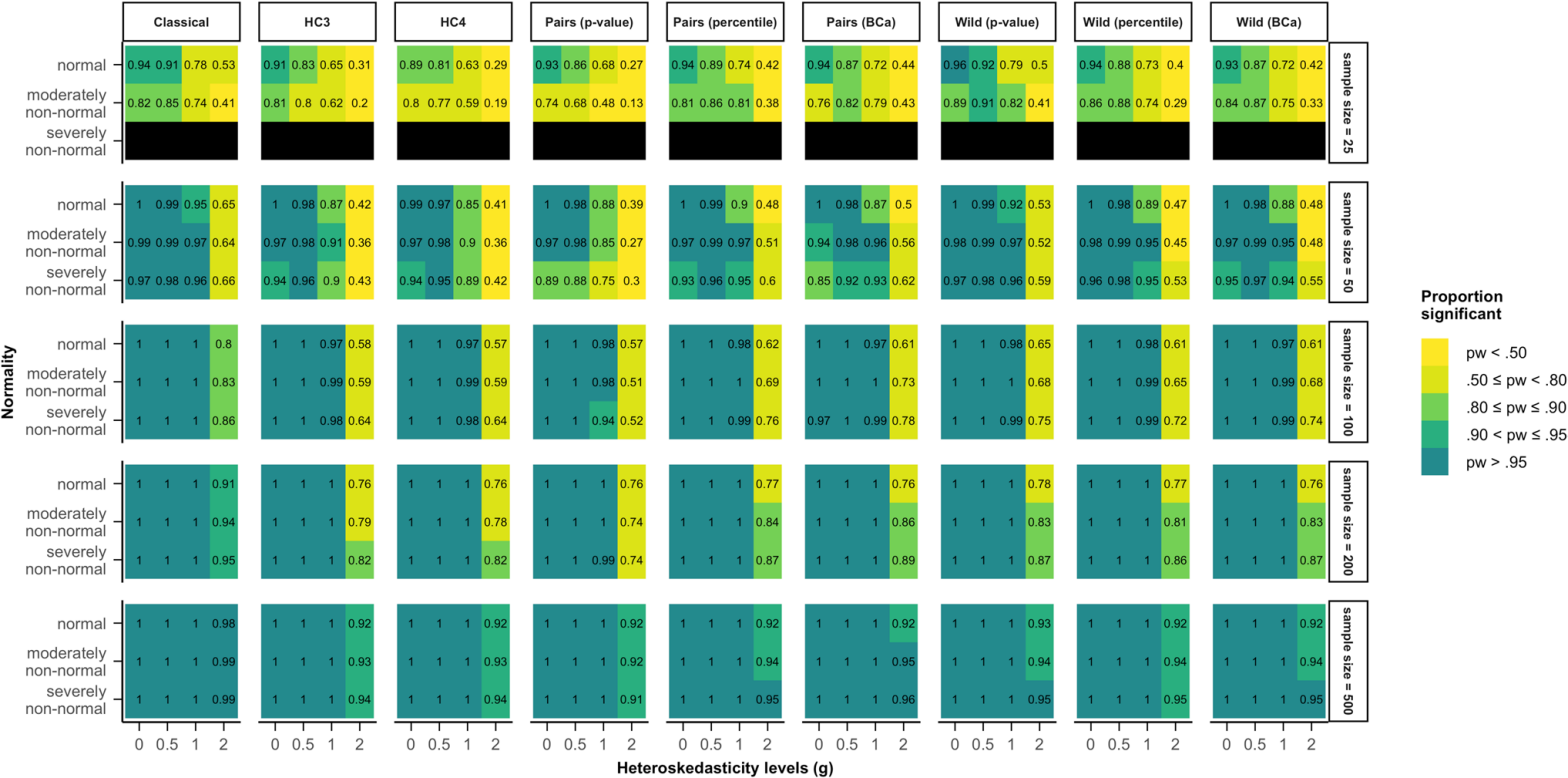
Statistical power of all tested methods and data scenarios, indicated by the proportion of significant results, with color-coded highlights depending on the reduction in power in model 2. *Note*. The simulated data was modeled after Eq. (6) (model 2), where heteroskedasticity was generated as a function of the predictor of interest (${x}_{1}$). *Classical*: classical inference typical for OLS regression, *HC3* & *HC4*: inference based on versions of the heteroskedasticity-consistent standard errors (Eqs. 11, 12), *Pairs*: pairs bootstrap resampling method, *Wild*: wild bootstrap resampling method (Eq. 14), p value: inference based on the bootstrap p value (Eq. 16), percentile: inference based on the bootstrap percentile confidence interval, BCa: inference based on the bias-corrected and accelerated bootstrap confidence interval

Figure 5 depicts the power estimates for all 9 methods and all data scenarios when the data was generated by model 2 (Eq. 6), where heteroskedasticity was a function of the predictor of interest. The classical inference method yielded a reduction in power with increasing heteroskedasticity. This reduction was most prevalent in samples of size 100 or smaller, where power could drop as low as 41% in samples of 25 cases. Contrary to heteroskedasticity, non-normality of the errors resulted only in a small reduction of power, which additionally could only be seen in samples of size 25 and 50. This pattern of stronger power decline due to heteroskedastic errors and lesser power decline due to non-normally distributed errors could also be seen in the other eight methods.

Compared to the classical method, the two HC standard errors yielded a slight reduction in power when errors were homoskedastic and normally distributed, and their reduction in power due to assumptions not being met was more pronounced. In the most severe heteroskedasticity condition, the HC methods were underpowered in samples as large as 200.

All bootstrap methods demonstrated approximately the same level of power as the classical method when the assumptions were met. As with the type I error rate results, the pairs bootstrap with the *p*-value was most similar to the HC standard error methods, showing the most severe reductions in power due to non-normality and heteroskedasticity of the errors. The wild bootstrap with the *p* value yielded the least amount of power reduction out of all robust methods, although it still did not reach a power of 80% in all conditions at a sample size of 200.

**Fig. 6:**
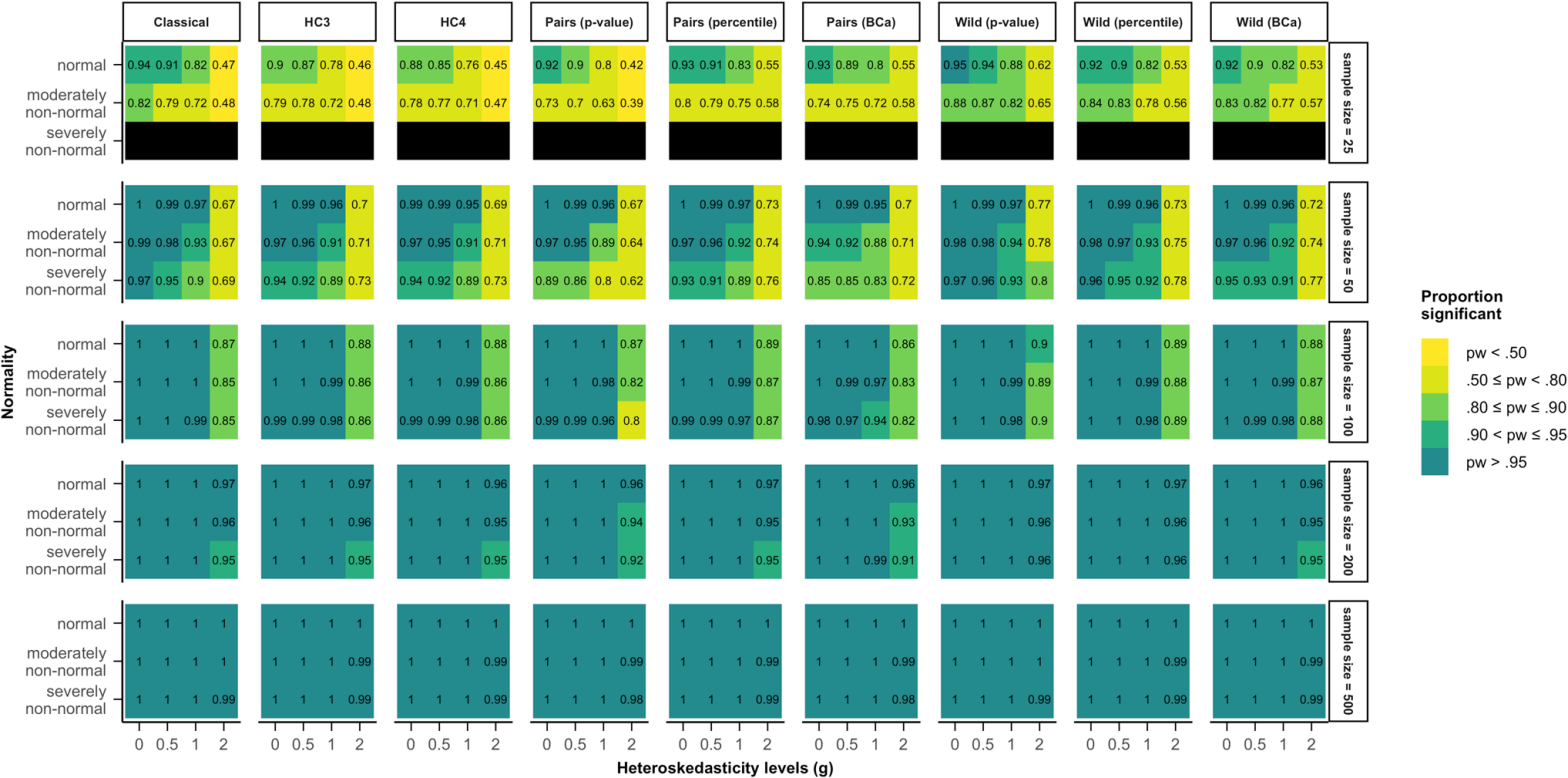
Statistical power of all tested methods and data scenarios, indicated by the proportion of significant results, with color-coded highlights depending on the reduction in power in model 4. *Note*. The simulated data was modeled after Eq. (8) (model 4), where heteroskedasticity was generated as a function of a different predictor (${x}_{2}$) not the predictor of interest (${x}_{1}$). Classical: classical inference typical for OLS regression, HC3 & HC4: inference based on versions of the heteroskedasticity-consistent standard errors (Eqs. 11, 12), Pairs: pairs bootstrap resampling method, Wild: wild bootstrap resampling method (Eq. 14), p value: inference based on the bootstrap p value (Eq. 16), percentile: inference based on the bootstrap percentile confidence interval, *BCa*: inference based on the bias-corrected and accelerated bootstrap confidence interval

In contrast, Fig. 6 shows the power estimate results for model 4 (Eq. 8). The pattern of the reduction in power due to either of the assumptions not being met was also reflected in the results when heteroskedasticity was a function of a different predictor. However, this pattern was less pronounced compared to when heteroskedasticity was a function of the predictor of interest. This time, the classical inference method, HC3, HC4 and the pairs bootstrap with the *p* value yielded very similar results: When errors were severely heteroskedastic power dropped under 50% at the smallest sample size, was between 50% and 80% at a sample size of 50, and was equal or larger than 80% for samples of 100 and up. One exception was the scenario with severe heteroskedastic and severely non-normally distributed errors at a sample size of 100, where the pairs bootstrap with the *p* value barely missed a power of 80%. Further, HC3 and HC4 again showed slightly worse power than the classical inference method when assumptions were met.

All other bootstrap methods, i.e., the pairs bootstrap with both confidence intervals and all of the wild bootstrap variations, performed similarly to each other. When assumptions were met, they yielded power estimates between 92% and 95% (compared to the classical method’s 94%). In samples of size 25 and 50, their power dropped under 80%, but never under 50%, when the errors were non-normally distributed or heteroskedastic. Both confidence interval versions of the pairs bootstrap declined slightly more in power when errors were non-normally distributed in the smallest sample size conditions. From a sample size of 100 onward, power was always above 80% for all five methods.

### Bias in the estimates

**Fig. 7:**
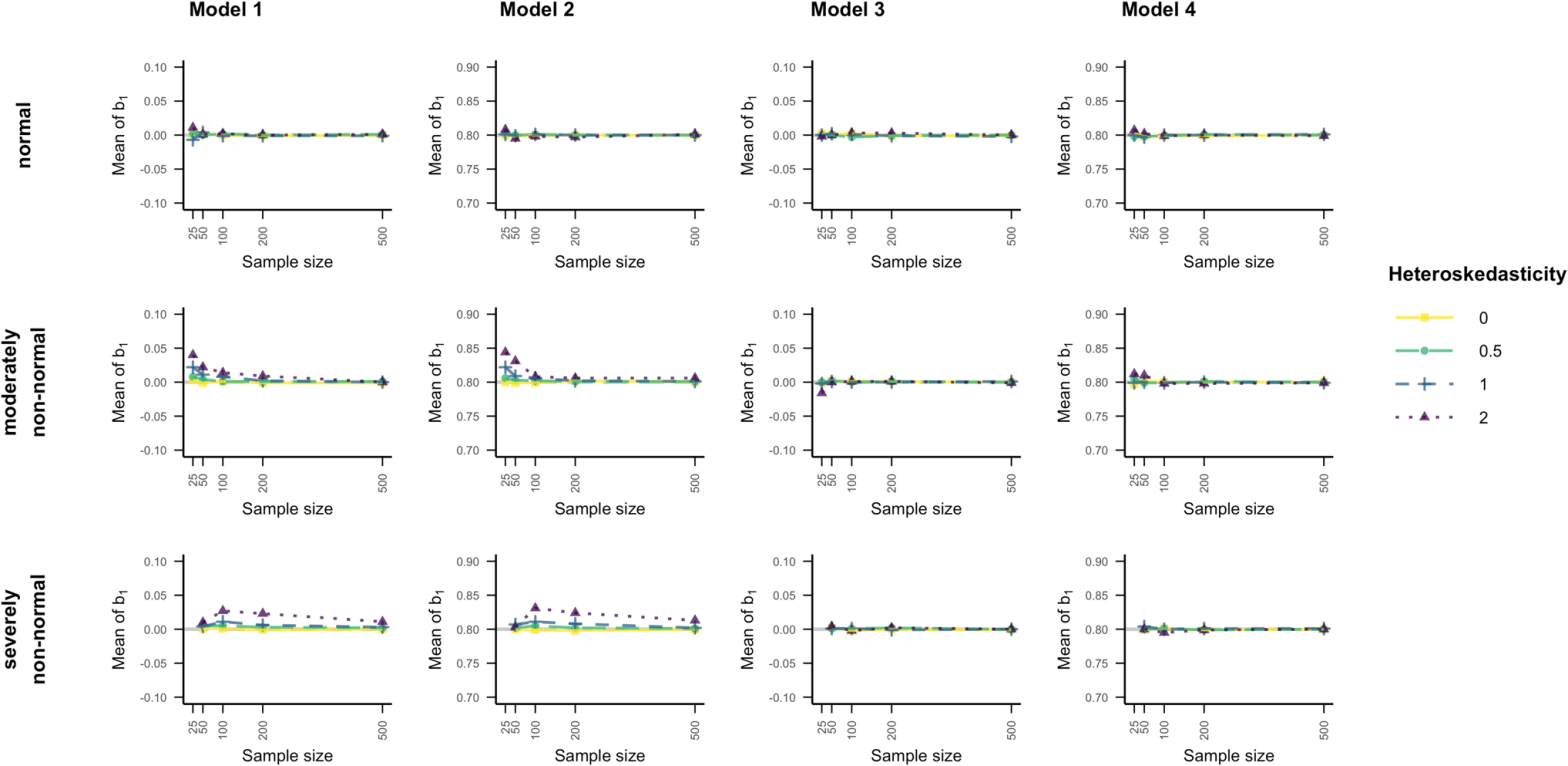
Deviation of the simulated OLS regression coefficient’s mean from the true parameter per heteroskedasticity level, normality level, sample size, and model. *Note*. ${b}_{1}$ denotes the regression coefficient of the predictor of interest (${x}_{1}$) in each model. Each mean was computed for the ${b}_{1}$ coefficient in the 10,000 regression models fit to the simulated data sets for the respective scenario. The data-generating models are described in Eqs. (5-8)

As noted in the introduction, even with violations of the homoskedasticity and normality assumptions, the estimates of the regression coefficients will be unbiased, i.e., the expected value of the $\widehat{{\boldsymbol{\beta}}}$ will be equal to the true parameter ${\boldsymbol{\beta}}$ (Berry, 1993; Cribari-Neto, 2004). However, in simulation, only a finite number of samples can be created, so even though the mean of all estimated regression coefficients of the effect of interest is expected to approximate the true parameter, slight deviations are to be expected. Figure 7 presents the means of the simulated OLS regression coefficients of the first predictor (${x}_{1}$) per data-generating model and scenario, which should be approximately 0 (in models 1 and 3) or 0.8 (in models 2 and 4). In small samples, a slight average bias of the estimated regression parameter was detectable if assumptions were not met.

To disentangle the effects of bias in the parameter and bias in the standard error, which both play a role in the proportion significant and coverage rates, the standard error bias was assessed separately. This could only be done for the three methods (classical inference, HC3, and HC4) that utilize a standard error for the computation of a test statistic in the significance test. All bootstrap methods covered here directly relied on the distributions of the bootstrapped regression coefficient estimates and thus did not compute standard errors.

To visualize bias in the respective methods’ standard errors, line plots were created to compare their sizes to the simulated standard error. The latter is represented by the standard deviation of the distribution of the simulated regression coefficients, i.e., a distribution of 10,000 estimated regression coefficients per scenario and model.

**Fig. 8:**
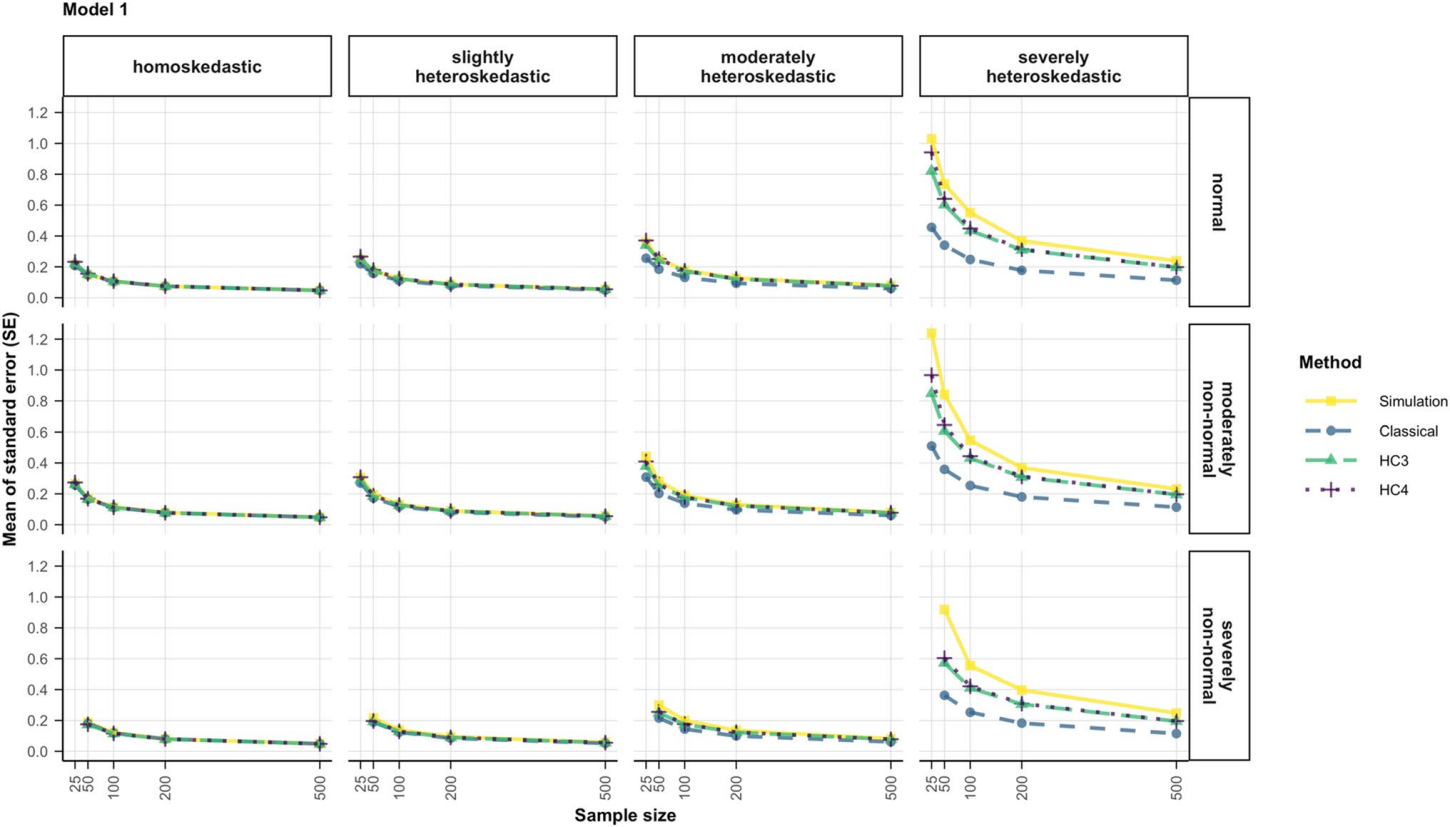
Comparison of the estimated standard errors (classical, HC3, HC4) to the standard deviation of the simulated distribution of regression coefficients for predictor ${x}_{1}$ for all the data scenarios in model 1. *Note*. The simulated data was modeled after Eq. (5) (model 1), where heteroskedasticity was generated as a function of the predictor of interest (${x}_{1}$). *Simulation*: the standard deviation of the simulated distribution of regression coefficients, which is a proxy for the “true standard error”, *Classical*: classical standard error typical for OLS regression, *HC3* & *HC4*: heteroskedasticity-consistent standard errors (Eqs. 11, 12)

When looking at the pattern of the simulated standard errors, Fig. 8 shows that more severe violations of homoskedasticity led to simulated sampling distributions with a larger variance, thus resulting in large simulated standard errors in these conditions (solid yellow line). Increasing deviations from normality hardly influenced the size of the simulated standard error when the errors were homoskedastic and led to modest increases in size when the errors were heteroskedastic. Lastly, smaller sample sizes expectedly showed larger simulated standard errors. A method of inference performs well if its estimated standard error matches this pattern.

When heteroskedasticity was severe, the classical standard error and the standard errors of HC3 and HC4 were consistently smaller than the simulated standard error. In general, HC3 and HC4 were very similar in their standard error estimation, but in small samples HC4 estimated a standard error slightly closer to the simulated standard error than HC3. In all sample sizes, the classical standard error consistently underestimated the simulated standard error.

This pattern was essentially the same in both models, where the heteroskedasticity was a function of the predictor of interest (model 1 and model 2). The plot for model 2 is not included here but can be found in the appendix (Fig. 15).

**Fig. 9:**
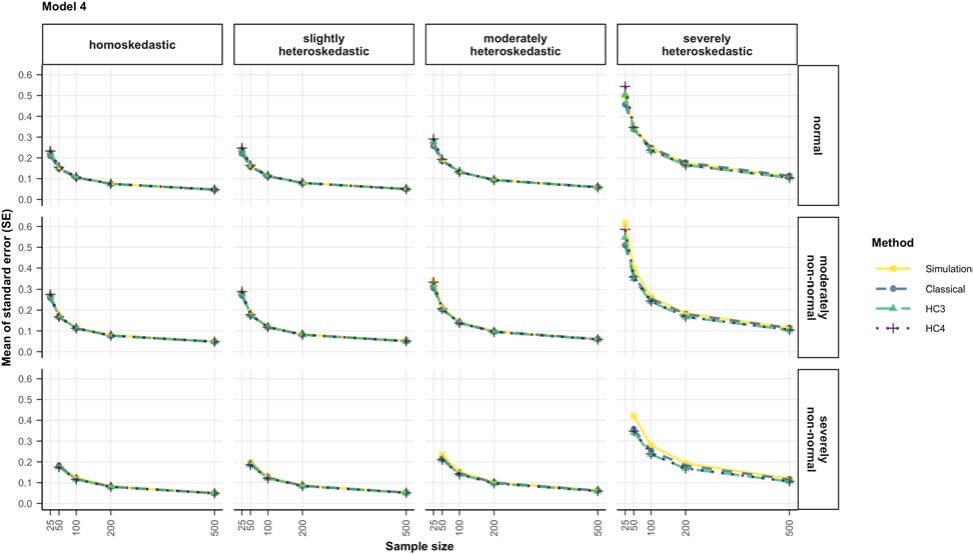
Comparison of the estimated standard errors (classical, HC3, HC4) to the standard deviation of the simulated distribution of regression coefficients for predictor ${x}_{1}$ for all the data conditions in model 3. *Note*. The simulated data was modeled after Eq. (7) (model 3), where heteroskedasticity was generated as a function of a different predictor (${x}_{2}$) not the predictor of interest (${x}_{1}$). *Simulation*: the standard deviation of the simulated distribution of regression coefficients, which is a proxy for the “true standard error”, *Classical*: classical standard error typical for OLS regression, *HC3* & *HC4*: heteroskedasticity-consistent standard errors (Eqs. 11, 12)

In models 3 and 4, where heteroskedasticity was a function of the other predictor, the average misestimation was rather small (see Fig. 9 for model 3, and Fig. 16 in the appendix for model 4). When the errors were severely heteroskedastic, and normal or moderately non-normal the classical standard error still showed the smallest estimate on average, but the difference was not as large as for models 1 and 2.

### Differences in the test statistic distributions

Another related reason why type I error or coverage rates diverge from the nominal alpha (or 1 – alpha) level is that the distribution of the test statistic does not follow the assumed *t*-distribution. For instance, in a model where the null hypothesis is true, i.e., the population regression coefficient is zero, a standard error that is estimated as too small will result in a test statistic ($t= b/{SE}_{b}$) that is too large. Such misestimated test statistics, in samples that violate assumptions, will thus likely not follow the *t*-distribution, which they are assumed to follow in NHST, given that the null hypothesis is true. For the visual analysis of the test statistic distributions, only the most problematic sample conditions (small samples with non-normally distributed and heteroskedastic errors) were chosen.

**Fig. 10:**
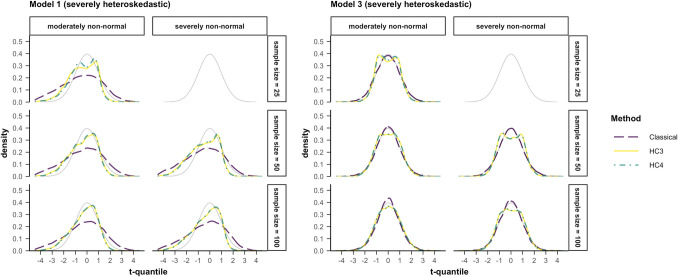
Distribution of the test statistics computed in data scenarios with severe violations of the normality and homoskedasticity assumption in model 1 and model 3. *Note*. The data-generating models 1 and 3 are described by Eqs. (5) and (7). The black dashed line indicates the true t-distribution with the respective degrees of freedom. The colored lines indicate the distributions of the 10,000 test statistics for each respective method, model and data scenario. *Classical*: classical test statistic typical for OLS regression, *HC3* & *HC4*: test statistic computed by using the heteroskedasticity-consistent standard errors (Eqs. 11, 12)

**Fig. 15:**
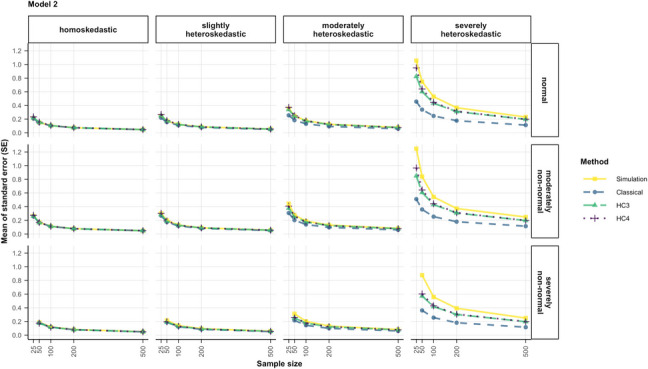
Comparison of the estimated standard errors (classical, HC3, HC4) to the standard deviation of the simulated distribution of regression coefficients for predictor ${x}_{1}$ for all the data scenarios in model 2. *Note*. The simulated data was modeled after Eq. (6) (model 2), where heteroskedasticity was generated as a function of the predictor of interest (${x}_{1}$). *Simulation*: the standard deviation of the simulated distribution of regression coefficients, which is a proxy for the “true standard error”, *Classical*: classical standard error typical for OLS regression, *HC3* & *HC4*: heteroskedasticity-consistent standard errors (Eqs. 11, 12)

**Fig. 16:**
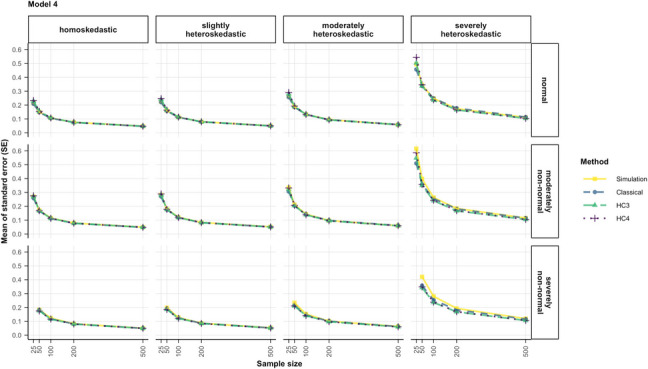
Comparison of the estimated standard errors (classical, HC3, HC4) to the standard deviation of the simulated distribution of regression coefficients for predictor ${x}_{1}$ for all the data conditions in model 4. *Note*. The simulated data was modeled after Eq. (8) (model 4), where heteroskedasticity was generated as a function of a different predictor (${{\boldsymbol{x}}}_{2}$) not the predictor of interest (${{\boldsymbol{x}}}_{1}$). *Simulation*: the standard deviation of the simulated distribution of regression coefficients, which is a proxy for the “true standard error”, *Classical*: classical standard error typical for OLS regression, *HC3* & *HC4*: heteroskedasticity-consistent standard errors (Eqs. 11, 12)

Figure 10 shows the different distributions of the test statistics computed by classical inference, HC3 and HC4 (dashed purple, solid yellow and dash-dotted cyan line) and the t-distribution centered around zero (thin grey line). In model 1 the distribution of a test statistic computed by the classical inference method has heavier tails than the t-distribution in the same scenarios. This means that values farther from zero are assumed to be less likely in the significance test with the classical method than they truly are. Thus, more than alpha percent (here 5%) of estimated test statistics (or empirical t-values) are being classified as “unlikely under the null”, which results in elevated rejection rates in OLS.

As seen in Fig. 10 the distribution of the test statistics for the same scenarios looked different when heteroskedasticity was not a function of the predictor of interest (model 3). In model 3 it becomes apparent why analyzing both the average bias in the standard error as well as the distribution of the test statistic is important. On average the standard errors in model 3, estimated by the classical method, HC3 and HC4, were not far off from the simulated standard error (Fig. 9). The rejection rates of the classical method match this pattern since they were very close to alpha. On the other hand, HC3 and HC4 were too conservative in small samples when errors were non-normally distributed and heteroskedastic (Fig. 2). Looking at the distributions of test statistics one can see that the ones resulting from using HC3 and HC4 (solid yellow and dash-dotted cyan line) have lighter tails than the *t*-distribution they are assumed to follow (thin grey line). The significance test, will therefore rate a test-statistic estimated by HC3 or HC4 as more likely under the null than it is under the true distribution of the test statistic, leading to less than alpha percent (5%) of test statistics being rejected. It should be noted that this only happens when the sample size is small (≤ 50 cases) and the errors are severely non-normally distributed. The larger the sample size gets, the closer the tails of the test statistic distribution approximate those of the* t*-distribution.

One explanation for this phenomenon could be the computation of the HC standard errors and their weighting of leverage values. Both HC methods estimate larger standard errors when high leverage values are present. If the cases with high leverage values have an impact on the effect of interest, this helps to somewhat attenuate the type I error rate. If, however, heteroskedasticity is a function of a different predictor, some cases could be high leverage cases for the whole regression model, but not necessarily for the effect of interest. In these instances, when the effect of interest has a sample estimate that is significantly larger or smaller than zero, the estimated HC standard errors are too large and result in a test statistic closer to zero. The consequence is a light-tailed distribution, where test statistic values accumulate slightly above and below zero.

## Discussion

The objective of this simulation study was to evaluate the performance of classical OLS regression in comparison with easily accessible and well-known alternative modes of inference in order to aid applied researchers on which methods are preferable when classical statistical inference assumptions are not satisfied. The considered simulated data samples were generated with varying deviations from normally distributed errors and violations of homoskedasticity in linear regression models. Five different sample sizes from 25 to 500 cases were examined as well as the impact of the source of heteroskedasticity, meaning whether it came from the predictor whose effect is analyzed or from a different (correlated) predictor. In all data scenarios, the classical inference method was compared to two methods that use heteroskedasticity-consistent standard errors (HC3 and HC4) and six bootstrap techniques. These techniques differed in their resampling methods, specifically, the pairs bootstrap or wild bootstrap, as well as their inferential approach, which included the bootstrap *p* value, percentile confidence interval, or bias-corrected and accelerated (BCa) confidence interval. The performance of each method was evaluated based on type I error rates, power estimates, and confidence interval coverage rates. For those methods that compute a standard error for significance testing, bias in the standard error was also examined.

### Performance of classical statistical inference

When the data were homoskedastic, the classical inference method showed rejection rates close to the alpha level, and coverage rates around 1 – alpha, even when the error distribution deviated from normality. The decline in statistical power due to non-normality, decreasing from around 94% to 82% in samples of 25 cases and even less in larger samples, was amongst the smallest compared to the other methods. However, it is important to note that this does not imply that the classical method is generally robust against violations of the normality assumption in samples of 25 and up. There exists a variety of other distributions, not covered in this study, that can exert varying effects on the type I error rate and power. Especially heavy-tailed distributions can lead to ample reductions in power, and skewness can increase type I error rates substantially (Field & Wilcox, 2017; Wilcox, 2022; Wilcox et al., 2013).

In case of heteroskedastic errors, classical inference was generally associated with reduced power. Additionally, heteroskedasticity had a large impact on type I error rates, as they could turn out as high as 26% regardless of sample size. This is in line with the literature (Astivia & Zumbo, 2019; Cribari-Neto, 2004; Long & Ervin, 2000), repeatedly reporting heteroskedasticity to substantially affect type I error rates.

Our present work further showed that the impact of heteroskedasticity was different, whether it was a function of the predictor of interest or a different correlated predictor. In the latter scenario, the type I error rate for the effect of interest of the classical inference method hardly shows any deviations from the nominal alpha level (5%), no matter the amount of heteroskedasticity or non-normality. This is in line with results from Long and Ervin (2000), who demonstrated that the rejection rates using classical inference were closest to the nominal alpha level if the examined coefficient was barely affected by heteroskedasticity. Unfortunately, in practice, noticing such a case could be challenging, because this entails that in bivariate partial scatter plots a discernible pattern of non-constant variance are observable not for the predictor of interest but rather for the other predictor. Conversely, the present study showed that in data situations where heteroskedasticity is not linked to the predictor of interest, the power of the classical method is still reduced in the presence of non-normally distributed errors and heteroskedasticity.

Examining the coverage rates paints a picture similar to the type I error rates. The confidence interval based on the classical method yields lowered coverage rates in data situations where heteroskedasticity, coming from the predictor of interest, is high.

In contrast, when heteroskedasticity was a function of a different predictor, the method performed adequately no matter the assumption violation, upholding rejection and coverage rates closest to the nominal alpha (or 1 – alpha) level when compared to the other robust techniques.

Deviations from the nominal alpha level or coverage rate, when heteroskedasticity is a function of the predictor of interest, can in part be explained by the misestimation of the classical standard error in scenarios in which the necessary assumptions are not met. In these scenarios, assessing the variance (or standard deviation) of the distribution of the 10,000 simulated estimated regression coefficients showed that these parameter estimates fluctuated considerably. The standard error of a regression coefficient is intended to be an estimate of this variability, specifically in the present study, an estimate of the simulated standard error. The classical method, typical for OLS regression, underestimates this variability greatly by computing standard errors that are too small, resulting in a rejection rate exceeding the nominal value and overly narrow confidence intervals. Additionally, the distributions of the t-values computed by the classical method follow a distribution with heavier tails than the theoretical t-distribution when assumptions are met (see Fig. 10), especially in scenarios with severe heteroskedasticity.

### Performance of conventional correction methods

#### Assumptions are met

Since the performance of classical inference is vastly different depending on the specific data situation, the question arises if there is a conventional correction method within the linear model framework that would generally be preferable.

If all assumptions for the classical inference are met, using either heteroskedasticity-consistent standard error (HC3 or HC4) keeps the rejection rate at approximately 5%. The power of the HC standard error methods in these instances is similar to the classical method, although HC4 seems to have marginally less power in small samples.

Regarding the bootstrap, there was a large variability in performance depending on the bootstrap resampling method (pairs versus wild), as well as the chosen inference method (bootstrap *p* value, percentile confidence interval, BCa confidence interval). When all assumptions were met, every bootstrap method seemed to require a large enough sample (200 cases or more) in order to keep the type I error rate at the nominal alpha (5%) level within the stringent criterion (Bradley, 1978). Only the wild bootstrap with the percentile confidence interval also showed rejection rates of around 5% in samples of 50 and 100 cases. In samples of size 25, the largest rejection rates of 10%, even though assumptions were met, were found using the wild bootstrap with a bootstrap *p* value. Hence, we advise against using any bootstrap method as the default, without checking if assumptions are even violated, specifically in smaller samples.

#### Assumptions are not met

If assumptions are not met, trying to identify the type and pattern of the violated assumptions is equally important. In the considered scenarios in the present study, it turned out that when heteroskedasticity was a function of the predictor of interest, HC3 and HC4 mostly performed satisfactorily for a variety of sample sizes and data situations regarding the type I error and coverage rates. Particularly, when it was only the heteroskedasticity assumption being violated, rejection rates were close to the nominal level for most scenarios, no matter the source of heteroskedasticity. This corresponds to what is reported in the literature (Cribari-Neto, 2004; Long & Ervin, 2000). Only when strong heteroskedasticity was combined with non-normality, both methods showed inflated rejection rates when heteroskedasticity was a function of the predictor of interest. This inflation decreased with larger sample sizes. In contrast, when heteroskedasticity was a function of a different correlated predictor, the rejection rates of the predictor of interest were too conservative in small samples with non-normally distributed errors. Unsurprisingly, the coverage rates showed a similar pattern: Depending on the source of heteroskedasticity the intervals are too narrow in data situations where both assumptions are violated, or too broad when the distribution deviates from normality. In either case, the performance gets better the larger the sample size. In terms of power, HC3 and HC4 perform similarly to OLS even when assumptions are not met, although the reduction in power due to heteroskedasticity is worse in both methods compared to OLS. This is the case no matter the source of heteroskedasticity.

Even though the performance regarding rejection rates, coverage rates and power hardly differs between HC3 and HC4, examining bias in the standard error showed some differences between the methods. In severely heteroskedastic situations, especially when the sample was small and errors were normally distributed, the HC4 standard error was slightly closer to the simulated standard error than the HC3 standard error. This difference between the HC methods was more obvious when the heteroskedasticity was a function of the predictor of interest and less so when it was a function of a different predictor. In fact, there was hardly any bias in both HC standard errors, when the heteroskedasticity was not a function of the predictor of interest. Here, the main problem is that in scenarios in which the errors deviated most strongly from normality and the sample size was 25 and 50, the distribution of the test statistics, computed with the HC standard errors, does not seem to follow a *t*-distribution (see Fig. 10). Instead, the tails appear relatively lighter, resulting in increased rejection rates which were reported above.

As with the data situations where assumptions were met, there was a large variability in performance of the different bootstrap methods when assumptions were not met. Regarding rejection rates, the pairs bootstrap with the bias-corrected and accelerated confidence intervals consistently performed the worst. It generally yielded rejection rates greater than 7.5% in almost all data scenarios, which only moderately improved with increasing sample size. The other bootstrap methods showed a much better performance when heteroskedasticity was a function of another predictor and not the predictor of interest. Here, the rejection rates of the pairs bootstrap with the percentile confidence interval and the wild bootstrap with the BCa confidence interval always stayed within 2.5% and 7.5%. The wild bootstrap with the percentile confidence interval also always stayed within the liberal criterion and even kept rejection rates within 4.5% and 5.5% in the majority of scenarios for samples of 100 cases and above. Using a bootstrap *p* value with the wild bootstrap resampling method showed rejection rates greater than 7.5% in small samples and using it with the pairs bootstrap resampling method yielded rejection rates smaller than 2.5% for non-normal errors in samples of up to 100 cases.

When heteroskedasticity was a function of the predictor of interest, the pairs bootstrap with a bootstrap *p* value showed results similar to the ones obtained with the two HC methods, regarding rejection rates. All other bootstrap methods showed elevated rejection rates, especially in samples of size 25, 50, and sometimes even 100 cases. After the pairs bootstrap with the *p* value, the wild bootstrap with percentile confidence intervals yielded rejection rates close to 5% in most data situations, although they were still outperformed by the two HC methods.

Regarding inference based on confidence intervals, out of the bootstrap methods, the wild bootstrap with the percentile confidence interval performed the most satisfactorily in both models, where the coverage rates were examined. Concerning power, all bootstrap methods performed comparably. The wild bootstrap with the bootstrap *p* value showed the least amount of power loss when assumptions were not met, although this was closely followed by both wild bootstrap with the percentile and the BCa confidence interval, and then the two pairs bootstrap confidence interval methods.

Overall, in most data situations examined here, the HC3/HC4 standard error methods or the wild bootstrap with the percentile confidence interval performed reasonably. According to previous research, HC4 might be preferable over HC3 in samples with large leverage values (Cribari-Neto, 2004), although no distinct performance differences were observed in this study.

For a clearer overview of all the various results of the present study, Tables 2 and 3 summarize them, simplifying them for type I error and coverage rate, and power, respectively. Grouped by sample size, Table 2 shows which methods perform satisfactorily according to the stringent or the liberal criterion and Table 3 does the same for power > 80% and power > 90%.

**Table 2:** Method performance regarding type I error and coverage rate

	Heteroskedasticity as function of the predictor of interest (model 1 & 2)				Heteroskedasticity as function of a different (correlated) predictor (model 3 & 4)			
Sample size	Homoskedastic & normal	Homoskedastic & non-normal	Heteroskedastic & normal	Heteroskedastic & non-normal	Homoskedastic & normal	Homoskedastic & non-normal	Heteroskedastic & normal	Heteroskedastic & non-normal
**25**	**Classical** **HC3** **HC4** Pairs *p* value Wild percentile Wild BCa	**Classical** Wild *p* value Wild percentile Wild BCa	HC3 HC4 Pairs *p* value	(HC4)	**Classical** **HC3** **HC4** Pairs *p* value Pairs percentile Pairs BCa Wild percentile Wild BCa	**Classical** **Wild BCa** Pairs percentile Wild *p* value Wild percentile	**Classical** Pairs percentile Wild percentile Wild BCa	Classical Pairs percentile Wild *p* value Wild percentile Wild BCa
**50**	**Classical** **HC3** **HC4** **Wild percentile** Pairs *p* value Pairs percentile Pairs BCa Wild *p* value Wild BCa	**Classical** Pairs percentile Wild *p* value Wild percentile Wild BCa	**HC4** HC3 Pairs *p* value	(HC3) (HC4) (Pairs *p* value)	**Classical** **HC3** **HC4** Pairs *p* value Pairs percentile Pairs BCa Wild *p* value Wild percentile Wild BCa	**Classical** Pairs percentile Wild *p* value Wild percentile Wild BCa	**Classical** HC3 HC4 Pairs percentile Wild *p* value Wild percentile Wild BCa	**Classical** Pairs percentile Wild *p* value Wild percentile Wild BCa
**100**	**Classical** **HC3** **HC4** **Pairs *p *value** **Wild percentile** **Wild BCa** Pairs percentile Pairs BCa Wild *p* value	**Classical** HC3 HC4 Pairs percentile Wild *p* value Wild percentile Wild BCa	**HC4** HC3 Pairs *p* value Pairs percentile Wild percentile	(HC3) (HC4) (Pairs *p* value)	**Classical** **HC3** **HC4** **Wild percentile** **Wild BCa** Pairs *p* value Pairs percentile Pairs BCa Wild *p* value	**Classical** **Wild percentile** HC3 HC4 Pairs percentile Wild *p* value Wild BCa	**Classical** HC3 HC4 Pairs *p* value Pairs percentile Wild *p* value Wild percentile Wild BCa	**Classical** **Wild percentile** HC3 HC4 Pairs percentile Wild *p* value Wild BCa
**200**	**All methods**	**Classical** **Wild percentile** HC3 HC4 Pairs *p* value Pairs percentile Wild *p* value Wild BCa	HC3 HC4 Pairs *p* value Pairs percentile Wild *p* value Wild percentile Wild BCa	(HC4) (Pairs *p* value)	**Classical** **HC3** **HC4** **Wild BCa** Pairs *p* value Pairs percentile Pairs BCa Wild *p* value Wild percentile	**Classical** **Wild percentile** HC3 HC4 Pairs *p* value Pairs percentile Wild *p* value Wild BCa	**Classical** **HC3** **HC4** **Wild percentile** Pairs *p* value Pairs percentile Wild *p* value Wild BCa	**Classical** HC3 HC4 Pairs *p* value Pairs percentile Wild *p* value Wild percentile Wild BCa
**500**	**All methods**	**Classical** **HC3** **HC4** **Pairs *p *value** Pairs percentile Pairs BCa Wild *p* value Wild percentile Wild BCa	**HC3** **HC4** **Pairs *p *value** **Wild percentile** Pairs percentile Pairs BCa Wild *p* value Wild BCa	HC3 HC4 Pairs *p* value	**All methods**	**Classical** **Wild *p *value** **Wild percentile** HC3 HC4 Pairs *p* value Pairs percentile Pairs BCa Wild BCa	**Classical** **HC3** **HC4** **Pairs *p *value** **Wild *p *value** **Wild percentile** Pairs percentile Pairs BCa Wild BCa	**Classical** **HC3** **HC4** Pairs *p* value Pairs percentile Wild *p* value Wild percentile Wild BCa

Bold text indicates that the method meets the stringent criterion. Unformatted text indicates that it met the liberal criterion (Bradley, 1978). If the text is set in round brackets, the method shows some problems but performs best among all tested methods. If all methods perform equally well, this is indicated by the shorthand notation “all methods” in the respective formatting. Rejection and coverage rates are reported for the regression coefficient of the predictor of interest. Heteroskedasticity can either be a function of this predictor or a function of a different (correlated) predictor. This only applies to data situations similar to the ones covered in this work

**Table 3:** Method performance regarding power

	Heteroskedasticity as function of the predictor of interest (model 2)				Heteroskedasticity as function of a different (correlated) predictor (model 4)			
Sample size	Homoskedastic & normal	Homoskedastic & non-normal	Heteroskedastic & normal	Heteroskedastic & non-normal	Homoskedastic & normal	Homoskedastic & non-normal	Heteroskedastic & normal	Heteroskedastic & non-normal
**25**	**Classical** **HC3** **Pairs *p* value** **Pairs percentile** **Pairs BCa** **Wild *p* value** **Wild percentile** **Wild BCa** HC4	Classical HC3 Pairs percentile Wild ***p*** value Wild percentile Wild BCa	(Classical) (Wild ***p*** value)	(Pairs percentile) (Wild ***p*** value)	**Classical** **Pairs *p* value** **Pairs percentile** **Pairs BCa** **Wild *p* value** **Wild percentile** **Wild BCa** HC3 HC4	Classical Wild ***p*** value Wild percentile Wild BCa	(Wild ***p*** value)	(Wild ***p*** value)
**50**	**All methods**	**Classical** **HC3** **HC4** **Pairs percentile** **Wild *p* value** **Wild percentile** **Wild BCa** Pairs ***p*** value Pairs BCa	(Classical) (Wild ***p*** value)	(Classical) (Pairs percentile) (Pairs BCa) (Wild ***p*** value)	**All methods**	**Classical** **HC3** **HC4** **Pairs percentile** **Wild *p* value** **Wild percentile** **Wild BCa** Pairs ***p*** value Pairs BCa	(All methods)	(All methods)
**100**	**All methods**	**All methods**	Classical	Classical	**All methods**	**All methods**	**Wild *p* value** Classical HC3 HC4 Pairs ***p*** value Pairs percentile Pairs BCa Wild percentile Wild BCa	Classical HC3 HC4 Pairs percentile Pairs BCa Wild ***p*** value Wild percentile Wild BCa
**200**	**All methods**	**All methods**	**Classical**	**Classical** Pairs percentile Pairs BCa Wild ***p*** value Wild percentile Wild BCa	**All methods**	**All methods**	**All methods**	**All methods**
**500**	**All methods**	**All methods**	**All methods**	**All methods**	**All methods**	**All methods**	**All methods**	**All methods**

Bold text indicates that the method achieved a power of > 90%. Unformatted text indicates that the power was > 80%. If the text is set in round brackets, the method does not (always) reach 80% power but performs best among all tested methods. If all methods perform equally well, this is indicated by the shorthand notation “all methods” in the respective formatting. Power is reported for the regression coefficient of the predictor of interest. Heteroskedasticity can either be a function of this predictor or a function of a different (correlated) predictor. This only applies to data situations similar to the ones covered in this work

### Sensitivity analysis

Even though the present simulation study can give some guidance on which methods to use under which circumstances, examining whether any given sample data comes from a population that violates the heteroskedasticity and/or the normality assumption is another thing entirely. As recommended in previous studies, significance tests should not be used when examining assumption violations, since their power may be too low to detect deviations in samples where violations actually matter (Field & Wilcox, 2017; Long & Ervin, 2000). We recommend first visualizing the sample data through partial scatter plots for each predictor and the residuals of the model to examine homoskedasticity and assessing histograms of the residuals (or p-p-plots or q-q-plots) for the distributional assumptions. Scatter plots that show a funnel, butterfly or inverse butterfly pattern (see Fig. 1 in Sladekova & Field, 2024d) could indicate problems with homoskedasticity, and histograms of the residuals depicting deviations from the normal curve shape could indicate non-normally distributed errors. However, this visual analysis is highly subjective and there are no clear guidelines to which extent deviations remain unproblematic. Recently, a method to quantify heteroskedasticity for continuous predictors was described by Sladekova and Field (2024d), which could provide a numerically descriptive addition to the visual analysis.

Generally, it is considered good practice to compare results from multiple methods, especially if the sample data suggests that assumptions are violated. If all methods agree, a conclusion could be seen as more sturdy (Wagenmakers et al., 2021). This consensus, obviously, does not eliminate the possibility that any conclusion drawn from the results is in fact a type I or type II error. It does, however, show that the reached conclusions are at least not sensitive to deviations from normality or heteroskedasticity, depending on the method used. If the conclusions differ depending on which method is used, knowledge about the type of assumption violation and the method’s performance can be used to evaluate the validity of a given result.

For instance, imagine a study with a sample of 60 cases, where the histogram of the residuals looks fairly normal, but analyzing the partial scatter plot shows a noticeable cone-shaped pattern around the regression line for the predictor of interest. Even though the assumption about heteroskedasticity pertains to the population errors, not the sample residuals, it might be more reasonable not to just blindly assume homoskedasticity in this scenario. For this reason, the imaginary researcher decides to use the default SPSS bootstrap method (i.e., pairs bootstrap with a percentile confidence interval) and compares it to a *p* value computed with the HC4 standard error. The percentile pairs bootstrap confidence interval indicates rejecting the null-hypothesis, but the HC4 *p* value indicates not to reject the null hypothesis.

Figure 1 shows that for a comparable sample of size 50 and normally distributed but at least moderately heteroskedastic errors, a pairs bootstrap with a percentile CI is more prone to making a type I error (8–9%) than HC4 (5%). This result can also be gathered from Table 2, where it is shown that for the condition “heteroskedastic & normal” and a sample size of 50, only HC4 ranged within the stringent criterion. Additionally, Fig. 5 shows that for the same conditions the power estimates, could go as low as 41% for HC4 and 48% for the percentile bootstrap depending on the amount of heteroskedasticity. This is reflected in Table 3 since neither HC4 nor the pairs bootstrap with the percentile CI are listed as having adequate power (i.e., ≥.80). Given this, a researcher who is more concerned about avoiding a type I error than a type II error, could then decide to base their conclusions on the HC4 rather than the bootstrap results. Prioritizing the percentile bootstrap results over HC4 due to higher power (i.e., lower type II error rate) could certainly also be argued for, although this might be harder to justify because both analyses demonstrated fairly low power in our simulation. Generally, no conclusion should solely be based on a statical analysis but should instead always weigh the consequences of making either a type I or type II error besides other (theoretical) considerations (Benjamin et al., 2017).

### A priori power

When planning a study, it is also important to think about whether it is likely that the respective assumptions of the analysis are met in the investigated populations. The simulations for the regression models showed that a steep decline in statistical power can be expected simply due to not meeting the homoskedasticity assumption. For instance, where a large effect would have a 94% power to be detected in a sample of 25 when assumptions are met, this drops to 53% when the errors are severely heteroskedastic and 41% when, in addition, the errors are non-normally distributed. Well-known power analysis programs, like G*Power (Faul et al., 2007) or SPSS’ integrated power analysis, can only compute power for the case that model assumptions are met. Figures 5 and 6 can give some guidance as to which combinations of assumption violations have a strong impact on statistical power at which sample size. In these figures, researchers can compare the upmost left cell for the classical inference method, where the data was generated to be both normal and homoskedastic, for each sample size to all other cells in the respective block. A stronger decrease in power for a condition compared to when assumptions are met implies that more participants would need to be tested. To figure out exactly how many participants would be needed, power could be assessed via simulation instead of analytically with programs like R. This way, all types of assumption violations and the expected population effect size can be explicitly modelled (Green & MacLeod, 2016; Lakens & Caldwell, 2021; Zimmer et al., 2023).

## Limitations

The data scenarios simulated in this study are only a fraction of what researchers may encounter in practice. Thus, based on this study alone, it is not valid to generalize to all possible cases of non-normally distributed and/or heteroskedastic errors. For instance, the heteroskedastic pattern (funnel shape) simulated here is only one of many. It has been reported that the wild bootstrap and the HC standard errors can differ in performance depending on the variance pattern (Ng & Wilcox, 2009). For instance, the butterfly and funnel shapes tend to decrease confidence interval coverage rates below their nominal values, whereas the inverse-butterfly shape tends to exceed them (Sladekova & Field, 2024a). The funnel shape can be encountered when variables have a “lower bound of zero but no upper bound” (Cohen et al., 2003, p. 244). Another example is measurements with floor or ceiling effects, where the variance at one endpoint of the scale is attenuated.

Further, even though the distribution of the predictors is not a subject of the assumptions of OLS regression and its inference, it should be noted that in this study, only predictors with continuous normal distributions were simulated. The different error distributions that were simulated were based on previous simulation studies, which themselves based these skewness and kurtosis values on data sets from several community-based mental health and substance abuse studies (Curran et al., 1996). Still, the present results only cover deviations from normality that are potentially more extreme than typically encountered (Blanca et al., 2013) and do not consider other distributional shapes such as uniform, negative skewness, or symmetric but heavy-tailed.

The tables presented in this study should therefore rather be considered as some support for practical researchers to orient themselves on which methods could and which methods will likely not work as expected for their specific data situation. Generally, it is worth highlighting that all results of statistical inference carry uncertainty, and any conclusion should be drawn modestly, acknowledging remaining doubts and limitations transparently (Wagenmakers et al., 2021).

Lastly, the methods assessed in this study exclusively focus on different approaches to inference for the OLS estimator, not the estimation of the parameters themselves. Within a linear regression framework, robust regression methods that employ a different estimator may be better suited to address specific phenomena that influence the results, such as outliers (Wilcox, 2022). However, in the long run, reflecting on potential data-generating processes behind non-normal distributional shapes or heteroskedastic variance patterns may foster a better understanding of underlying psychological or domain-specific mechanisms. Once generative models reflecting such mechanisms can be formulated, they will probably soon surpass any simple linear model for epistemic purposes (that is, for gaining knowledge and understanding about real-world phenomena). However, using a robust inference method for an OLS regression can still be a helpful first attempt to make sense of some given data while still protecting against increased type I and/or type II error rates.

## Conclusion

The objective of the present study was to compare various robust alternatives available in SPSS (version 29) to the classical inference method for ordinary least squares regression analysis. A variety of data scenarios, created with differing sample sizes and levels of violation of the homoskedasticity and normality assumptions, were simulated based on four different data-generating models. The results showed that no method out of the classical method, HC3, HC4 and the six different bootstrap methods was found to perform satisfactorily on all accounts in the considered data scenarios in terms of type I error, coverage rates and power.

Either heteroskedasticity alone or in conjunction with non-normality led to the worst performances for most methods. The classical method frequently underestimated the size of the standard error, leading to overly narrow confidence intervals and grossly inflated type I error rates. Using either HC3 or HC4 standard errors, or a wild bootstrap procedure with percentile confidence intervals, led to decent results in many but not all considered data situations when considering type I error rate, coverage rate, as well as power. The present study aims to provide some guidance for applied researchers to find more suitable alternatives to the classical inference typical for OLS regression when their data cast doubt on the classical approach's validity. Generally, comparing multiple methods and investigating their differences may promote a more comprehensive understanding of one’s data and their underlying generating mechanisms or processes.

## Figures and Tables

**Fig. 11:**
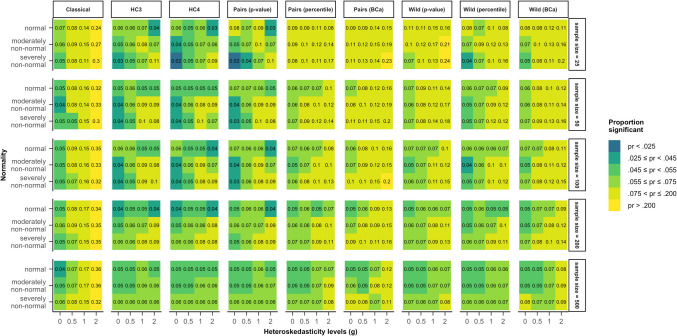
Under random sampling: Type I error rates for all tested methods and data scenarios with color-coded highlights depending on the deviation from the nominal alpha value (5%) in model 1 (heteroskedasticity as a function of the predictor of interest)

**Fig. 12:**
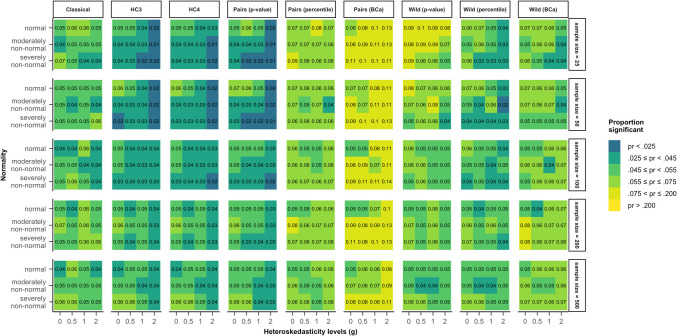
Under random sampling: Type I error rates for all tested methods and data scenarios with color-coded highlights depending on the deviation from the nominal alpha value (5%) in model 3 (heteroskedasticity as a function of a different predictor)

**Fig. 13:**
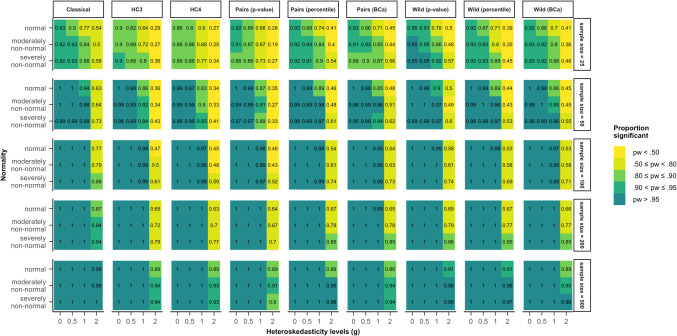
Under random sampling: Statistical power of all tested methods and data scenarios, indicated by the proportion of significant results, with color-coded highlights depending on the reduction in power in model 2 (heteroskedasticity as a function of a different predictor)

**Fig. 14:**
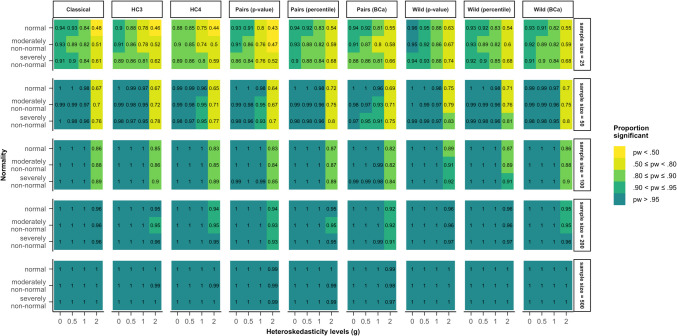
Under random sampling: Statistical power of all tested methods and data scenarios, indicated by the proportion of significant results, with color-coded highlights depending on the reduction in power in model 4 (heteroskedasticity as a function of a different predictor)

## Data Availability

The code for the simulation study is also available at the project’s Open Science Framework (OSF) page: https://osf.io/6c9u8/.

## Appendix

### BCa confidence interval computation

The BCa confidence interval of the $k$-th regression coefficient (with $k=0, \dots , K$) takes the shape of the bootstrap sampling distribution into account. It contains a parameter to correct for bias in the estimate (here denoted as $w$), along with an acceleration parameter (denoted as $a$) to correct for skew in the distribution.

The new corrected lower or upper confidence interval bound, depending on which value $\alpha$ takes on, for the $k$-th regression coefficient is given by ${\widetilde{\alpha }}_{k}$:

$$\begin{array}{l}{\widetilde{\alpha}}_{k}=\Phi \left({w}_{k}+\frac{{w}_{k}+{z}_{\alpha }}{1-{a}_{k}({w}_{k}+{z}_{\alpha})}\right)\\ {z}_{\alpha}={\Phi }^{-1}(\alpha)\end{array}$$

where $\Phi (\bullet )$ is the distribution function of a standard normal distribution ($N\sim (0, 1)$) where the result is a probability of a given z-quantile, and ${\Phi }^{-1}(\bullet )$ is the inverse of that, i.e. the quantile function, where the result is the z-quantile of a given probability. Here $\alpha$ denotes the chosen probability value for the lower (f.i., 0.025) or upper (f.i., 0.975) confidence interval bound.

The bias correction parameter is computed as:

$${w}_{k}={\Phi}^{-1}\left(\frac{1}{B+1}\left|\left\{\widehat{\beta}_{bk}^{\ast}\le\widehat{\beta}_{k}\right\}\right|\right)$$

And the acceleration parameter is computed as:

$${a}_{k}= \frac{1}{6}\frac{\sum_{i=1}^{n}{l}_{ik}^{3}}{{\left(\sum_{i=1}^{n}{l}_{ik}^{2}\right)}^{{~}^{3}\!\left/ \!{~}_{2}\right.}}$$

with the influence values of the $i$-th case and the $k$-th predictor:

$${l}_{ik}=\left(n-1\right)\ast({\widehat{\beta }}_{k}-{\widehat{\beta }}_{k,-i})$$

The computation of ${l}_{i}$ in SPSS closely follows the formulas described by Davison and Hinkley (1997). Other sources such as Fritz et al. (2012) describe a slightly different computation for the acceleration parameter a, where the influence value of the $i$-th case ${l}_{i}$ is not computed as a difference between the original estimate and the jackknife estimate (without the $i$-th case) but instead the difference between the mean of all jackknife estimates and the $i$-th jackknife estimate. To put it another way, other software packages or open-source R libraries may utilize similar but not identical computations for this method, while still using the same name for it.

For both the BCa and the percentile confidence interval SPSS uses interpolation if $\left(B+1\right)\alpha$, where $\alpha$ is either the probability of the lower or upper bound of the confidence interval, is not an integer. Say, all $B$ bootstrap estimates (of the $k$-th predictor) were lined up smallest to largest, then ${\widehat{\beta }}_{((B+1)\alpha )}^{\ast}$ is the bootstrap regression coefficient on the position $\left(B+1\right)\alpha$. If the latter is not an integer (e.g., $\left(1000+1\right)\ast\ 0.025=25.025$), the value indicating the lower bound of the bootstrap confidence interval would be the bootstrap regression coefficient on the position 25.025, which does not exist and some approximation to this value is needed. The linear interpolation on the normal quantile scale is given by:

$${\widehat{\beta}}_{((B+1)\alpha )}^{\ast}={\widehat{\beta }}_{(j)}^{\ast}+\frac{{\Phi}^{-1}\left(\alpha \right)-{\Phi }^{-1}\left(\frac{j}{B+1}\right)}{{\Phi }^{-1}\left(\frac{j+1}{B+1}\right)-{\Phi}^{-1}\left(\frac{j}{B+1}\right)}\left({\widehat{\beta}}_{(j+1)}^{\ast}-{\widehat{\beta}}_{(j)}^{\ast}\right)$$

where $j$ is the integer part of $\left(B+1\right)\alpha$. The interpolation fails if $k$ is $0$, $B$, or $B+1$. If this happens, SPSS quotes the extreme value and prints the implied level of error equal to $1/(B+1)$ (IBM, 2022).

### Random sampling versus restricted sampling

In order to compare the influence of restricting the sampling process versus random sampling on type I and II error rates, we simulated an additional 1000 data sets where the sampling process was strictly random (i.e., not restricted in any form). In the original simulation, samples were only retained if their error distribution resembled both population skewness and kurtosis. In this additional simulation, no such restriction was enforced. In the original simulation, the type I error rate of the classical inference method was hardly influenced by differences in normality, only by differences in heteroskedasticity. For methods like the heteroskedasticity-corrected standard errors, non-normality decreased type I error rate under homoskedasticity. These results were also found in our additional simulation, where the error distribution sampling process was not restricted, with the only difference that non-normality had less of an impact for HC3 and HC4 (see Figs. 11 and 12).

As in the original simulation, statistical power also decreased for increasing non-normality (increasing skewness and kurtosis) when sampling at random. Compared to the original simulation, this decrease was much less pronounced in the additional simulation (see Figs. 13 and 14). A possible explanation for this finding lies in the algorithm used to simulate a distribution with specific skewness and kurtosis values.

Simulating skewed and kurtotic distributions with the Vale and Maurelli/Fleishman method has previously been found to downwardly bias skewness and especially kurtosis (Astivia & Zumbo, 2015). In fact, we found that for random sampling, the mean skewness and kurtosis were, under homoskedasticity, lower than their population values and variability was substantial. For restricted sampling under homoskedasticity, variability was still high, but on average, the residual kurtosis was close to population kurtosis, and skewness was slightly overestimated on average.

Using g-and-h distributions, Wilcox (2022) has reported that for a fixed g (a proxy for skewness), increasing h (a proxy for kurtosis) decreases the type I error rate. Our results agree with Wilcox’s finding insofar as showing that when sampling at random (i.e., where kurtosis values on average are lower than for restricted sampling), power decreased less under increasing non-normality (higher skewness, higher kurtosis)

The results for the comparison of the estimated standard errors in model 2 and model 4 are shown in Figs. 15 and 16, respectively. These figures illustrate the differences in estimated standard errors compared to the simulated standard error.

## References

[CR1] Astivia, O. L. O., & Zumbo, B. D. (2015). A cautionary note on the use of the Vale and Maurelli method to generate multivariate, nonnormal data for simulation purposes. *Educational and Psychological Measurement,**75*(4), 541–567. 10.1177/0013164414548894 29795832 10.1177/0013164414548894 PMC5965614

[CR2] Astivia, O. L. O., & Zumbo, B. D. (2019). Heteroskedasticity in multiple regression analysis: What it is, how to detect it and how to solve it with applications in R and SPSS. *Practical Assessment, Research, and Evaluation*, *24*(1). 10.7275/Q5XR-FR95

[CR3] Benjamin, D. J., Berger, J. O., Johannesson, M., Nosek, B. A., Wagenmakers, E.-J., Berk, R., Bollen, K. A., Brembs, B., Brown, L., Camerer, C., Cesarini, D., Chambers, C. D., Clyde, M., Cook, T. D., De Boeck, P., Dienes, Z., Dreber, A., Easwaran, K., Efferson, C., …, & Johnson, V. E. (2017). Redefine statistical significance. *Nature Human**Behaviour*, *2*(1), 6–10. 10.1038/s41562-017-0189-z 10.1038/s41562-017-0189-z 30980045

[CR4] Berry, W. (1993). *Understanding regression assumptions*. SAGE Publications, Inc. eBooks. 10.4135/9781412986427

[CR5] Blanca, M. J., Arnau, J., López-Montiel, D., Bono, R., & Bendayan, R. (2013). Skewness and kurtosis in real data samples. *Methodology,**9*(2), 78–84. 10.1027/1614-2241/a000057

[CR6] Blanca, M. J., Alarcón, R., & Bono, R. (2018). Current practices in data analysis procedures in psychology: What has changed? *Frontiers in Psychology,**9*, 2558. 10.3389/fpsyg.2018.02558 30618979 10.3389/fpsyg.2018.02558 PMC6300498

[CR7] Bono, R., Blanca, M. J., Arnau, J., & Gómez-Benito, J. (2017). Non-normal distributions commonly used in health, education, and social sciences: A systematic review. *Frontiers in Psychology,**8*, 1602. 10.3389/fpsyg.2017.01602 28959227 10.3389/fpsyg.2017.01602 PMC5603665

[CR8] Bradley, J. V. (1978). Robustness? *British Journal of Mathematical and Statistical Psychology,**31*(2), 144–152. 10.1111/j.2044-8317.1978.tb00581.x

[CR9] Cohen, J. (1988). *Statistical Power Analysis for the Behavioral Sciences* (2nd ed.). Lawrence Erlbaum Associates.

[CR10] Cohen, J., Cohen, P., West, S. G., & Aiken, L. S. (2003). *Applied Multiple Regression/Correlation Analysis for the Behavioral Sciences* (3rd ed.). Lawrence Erlbaum Associates.

[CR11] Cribari-Neto, F. (2004). Asymptotic inference under heteroskedasticity of unknown form. *Computational Statistics & Data Analysis,**45*(2), 215–233. 10.1016/S0167-9473(02)00366-3

[CR12] Curran, P. J., West, S. G., & Finch, J. F. (1996). The robustness of test statistics to nonnormality and specification error in confirmatory factor analysis. *Psychological Methods,**1*(1), 16–29. 10.1037/1082-989X.1.1.16

[CR13] Davidson, R., & Flachaire, E. (2008). The wild bootstrap, tamed at last. *Journal of Econometrics,**146*(1), 162–169. 10.1016/j.jeconom.2008.08.003

[CR14] Davison, A. C., & Hinkley, D. V. (1997). *Bootstrap methods and their application*. Cambridge University Press.

[CR15] Faul, F., Erdfelder, E., Lang, A.-G., & Buchner, A. (2007). G*power 3: A flexible statistical power analysis program for the social, behavioral, and biomedical sciences. *Behavior Research Methods,**39*(2), 175–191. 10.3758/BF03193146 17695343 10.3758/bf03193146

[CR16] Field, A. P., & Wilcox, R. R. (2017). Robust statistical methods: A primer for clinical psychology and experimental psychopathology researchers. *Behaviour Research and Therapy,**98*, 19–38. 10.1016/j.brat.2017.05.013 28577757 10.1016/j.brat.2017.05.013

[CR17] Flachaire, E. (2005). Bootstrapping heteroskedastic regression models: Wild bootstrap vs. pairs bootstrap. *Computational Statistics & Data Analysis,**49*(2), 361–376. 10.1016/j.csda.2004.05.018

[CR18] Fleishman, A. I. (1978). A method for simulating non-normal distributions. *Psychometrika,**43*(4), 521–532. 10.1007/BF02293811

[CR19] Fritz, M. S., Taylor, A. B., & MacKinnon, D. P. (2012). Explanation of two anomalous results in statistical mediation analysis. *Multivariate Behavioral Research,**47*(1), 61–87. 10.1080/00273171.2012.640596 24049213 10.1080/00273171.2012.640596 PMC3773882

[CR20] Green, P., & MacLeod, C. J. (2016). SIMR: An R package for power analysis of generalized linear mixed models by simulation. *Methods in Ecology and Evolution,**7*(4), 493–498. 10.1111/2041-210X.12504

[CR21] Hayes, A. F., & Cai, L. (2007). Using heteroskedasticity-consistent standard error estimators in OLS regression: An introduction and software implementation. *Behavior Research Methods,**39*(4), 709–722. 10.3758/BF03192961 18183883 10.3758/bf03192961

[CR22] Hesterberg, T. (2011). Bootstrap. *Wiley Interdisciplinary Reviews: Computational Statistics,**3*(6), 497–526. 10.1002/wics.182

[CR23] IBM. (2022). IBM SPSS Statistics Algorithms. IBM Corporation. Retrieved September 1, 2025, from https://www.ibm.com/docs/en/SSLVMB_29.0.0/pdf/IBM_SPSS_Statistics_Algorithms.pdf

[CR24] Jones, L. V., & Tukey, J. W. (2000). A sensible formulation of the significance test. *Psychological Methods,**5*(4), 411–414. 10.1037/1082-989X.5.4.411 11194204 10.1037/1082-989x.5.4.411

[CR25] Knief, U., & Forstmeier, W. (2021). Violating the normality assumption may be the lesser of two evils. *Behavior Research Methods,**53*(6), 2576–2590. 10.3758/s13428-021-01587-5 33963496 10.3758/s13428-021-01587-5 PMC8613103

[CR26] Lakens, D., & Caldwell, A. R. (2021). Simulation-based power analysis for factorial analysis of variance designs. *Advances in Methods and Practices in Psychological Science,**4*(1), 2515245920951503. 10.1177/2515245920951503

[CR27] Long, J. S., & Ervin, L. H. (2000). Using heteroscedasticity consistent standard errors in the linear regression model. *The American Statistician,**54*(3), 217–224. 10.1080/00031305.2000.10474549

[CR28] MacKinnon, J. G. (2006). Bootstrap methods in econometrics. *Economic Record*, *82*(s1). 10.1111/j.1475-4932.2006.00328.x

[CR29] MacKinnon, J. G. (2013). Thirty Years of Heteroskedasticity-Robust Inference. In X. Chen & N. R. Swanson (Eds.), *Recent Advances and Future Directions in Causality, Prediction, and Specification Analysis* (pp. 437–461). Springer New York. 10.1007/978-1-4614-1653-1_17

[CR30] Micceri, T. (1989). The unicorn, the normal curve, and other improbable creatures. *Psychological Bulletin,**105*(1), 156–166. 10.1037/0033-2909.105.1.156

[CR31] Nevitt, J., & Hancock, G. R. (2001). Performance of Bootstrapping Approaches to Model Test Statistics and Parameter Standard Error Estimation in Structural Equation Modeling. *Structural Equation Modeling: A Multidisciplinary Journal, 8*(3), 353–377. 10.1207/S15328007SEM0803_2

[CR32] Ng, M., & Wilcox, R. R. (2009). Level robust methods based on the least squares regression estimator. *Journal of Modern Applied Statistical Methods,**8*(2), 384–395. 10.22237/jmasm/1257033840

[CR33] Pek, J., Wong, O., & Wong, A. C. M. (2018). How to address non-normality: A taxonomy of approaches, reviewed, and illustrated. *Frontiers in Psychology,**9*, 2104. 10.3389/fpsyg.2018.02104 30459683 10.3389/fpsyg.2018.02104 PMC6232275

[CR34] Robitzsch, A., & Grund, S. (2024). *miceadds: Some Additional Multiple Imputation Functions, Especially for “mice”* (Version R package version 3.17-44) [Computer software]. Retrieved September 1, 2025, from https://CRAN.R-project.org/package=miceadds

[CR35] Shear, B. R., & Zumbo, B. D. (2013). False positives in multiple regression: Unanticipated consequences of measurement error in the predictor variables. *Educational and Psychological Measurement,**73*(5), 733–756. 10.1177/0013164413487738

[CR36] Sladekova, M., & Field, A. P. (2024a). *Commonly Used Statistical Models in Psychology are Not Equipped to Deal with Real-World Conditions: A Simulation Study*. 10.31234/osf.io/xb4at

[CR37] Sladekova, M., & Field, A. P. (2024b). *In Search of Unicorns: Assessing Statistical Assumptions in Real Psychology Datasets*. 10.31234/osf.io/4rznt

[CR38] Sladekova, M., & Field, A. P. (2024c). *Psychology Researchers’ Self-Reported Knowledge of Sources of Bias in General Linear Models and**How**it Affects Their Analytic Practice*. 10.31234/osf.io/uhswb

[CR39] Sladekova, M., & Field, A. P. (2024d). *Quantifying Heteroscedasticity in Linear Models Using Quantile LOWESS Intervals*. 10.31234/osf.io/gn4mr 10.1037/met0000821 42095868

[CR40] Torres, A. F. C., & Akbaritabar, A. (2024). The use of linear models in quantitative research. *Quantitative Science Studies,**5*(2), 426–446. 10.1162/qss_a_00294

[CR41] Wagenmakers, E.-J., Sarafoglou, A., Aarts, S., Albers, C., Algermissen, J., Bahník, Š, Van Dongen, N., Hoekstra, R., Moreau, D., Van Ravenzwaaij, D., Sluga, A., Stanke, F., Tendeiro, J., & Aczel, B. (2021). Seven steps toward more transparency in statistical practice. *Nature Human Behaviour,**5*(11), 1473–1480. 10.1038/s41562-021-01211-8 34764461 10.1038/s41562-021-01211-8

[CR42] Wilcox, R. R. (2022). *Introduction to Robust Estimation and Hypothesis Testing* (5th ed.). Academic Press.

[CR43] Wilcox, R. R., Carlson, M., Azen, S., & Clark, F. (2013). Avoid lost discoveries, because of violations of standard assumptions, by using modern robust statistical methods. *Journal of Clinical Epidemiology,**66*(3), 319–329. 10.1016/j.jclinepi.2012.09.003 23195918 10.1016/j.jclinepi.2012.09.003

[CR44] Williams, M. N., Grajales, C. A. G., & Kurkiewicz, D. (2013). Assumptions of multiple regression: Correcting two misconceptions. *Practical Assessment, Research, and Evaluation*, *18*(11). 10.7275/55hn-wk47

[CR45] Zimmer, F., Henninger, M., & Debelak, R. (2023). Sample size planning for complex study designs: A tutorial for the mlpwr package. *Behavior Research Methods,**56*(5), 5246–5263. 10.3758/s13428-023-02269-0 38030925 10.3758/s13428-023-02269-0 PMC11289223

